# Alternative mechanisms of Notch activation by partitioning into distinct endosomal domains

**DOI:** 10.1083/jcb.202211041

**Published:** 2024-02-15

**Authors:** Hideyuki Shimizu, Samira Hosseini-Alghaderi, Simon A. Woodcock, Martin Baron

**Affiliations:** 1 School of Biological Sciences, Manchester Academic Health Science Centre, University of Manchester, Manchester, UK

## Abstract

Different membrane microdomain compositions provide unique environments that can regulate signaling receptor function. We identify microdomains on the endosome membrane of *Drosophila* endosomes, enriched in lipid-raft or clathrin/ESCRT-0, which are associated with Notch activation by distinct, ligand-independent mechanisms. Transfer of Notch between microdomains is regulated by Deltex and Suppressor of deltex ubiquitin ligases and is limited by a gate-keeper role for ESCRT complexes. Ubiquitination of Notch by Deltex recruits it to the clathrin/ESCRT-0 microdomain and enhances Notch activation by an ADAM10-independent/TRPML-dependent mechanism. This requirement for Deltex is bypassed by the downregulation of ESCRT-III. In contrast, while ESCRT-I depletion also activates Notch, it does so by an ADAM10-dependent/TRPML-independent mechanism and Notch is retained in the lipid raft-like microdomain. In the absence of such endosomal perturbation, different activating Notch mutations also localize to different microdomains and are activated by different mechanisms. Our findings demonstrate the interplay between Notch regulators, endosomal trafficking components, and Notch genetics, which defines membrane locations and activation mechanisms.

## Teaser

Sorting of signaling receptors between membrane environments can regulate their activity. Shimizu et al. show that localization of Notch to alternative microdomains on the endosome switches between different mechanisms of activation, and lateral movement of Notch on the endosome membrane between these microdomains is regulated by Deltex and limited by differential roles of ESCRT-I and III complexes.

## Introduction

Developmental signaling pathways need strict regulation to avoid pathological consequences. Precise control of membrane protein localization within different membrane microdomains can control signaling activity. However, there is still much that is not understood regarding how the movement of membrane proteins between membrane microdomains is restricted or regulated. Flat clathrin lattices have been identified as one such microdomain present on the early endosome (Raposo et al., 2001; Raiborg et al., 2001, 2006). The role of this microdomain is unclear but it may help to recruit proteins away from recycling pathways and toward lysosomal degradation (Raposo et al., 2001; Sachse et al., 2002). Clathrin recruits endosomal sorting complexes required for transport (ESCRT)-0 component Hrs to the localized microdomain (Raiborg et al., 2006). Hrs is a ubiquitin-binding protein that, along with signal-transducing adaptor molecule (STAM), recruits ubiquitinated cargo molecules for transfer to intraluminal vesicles (ILV) by the actions of the ESCRT-I to III complexes (reviewed by Frankel and Audhya [2018]). ESCRT-I and II complexes concentrate cargo, initiate membrane bending, and recruit ESCRT-III components. Clathrin can promote cargo dissociation from ESCRT-0 and transfer to ESCRT-I (Wenzel et al., 2018). ESCRT-III promotes pinching off of the neck of the budding ILV, releasing it into the endosomal lumen (Williams and Urbé, 2007; Boura et al., 2012a; Wollert and Hurley, 2010). VPS4 activity is required for disassembly of the ESCRT-III complex components for subsequent reuse (Han and Hill, 2019) and may also contribute to the ILV neck-pinching process (Adell et al., 2014).

Notch is a conserved, membrane-spanning, developmental signaling receptor with pleiotropic roles in cell fate regulation across many tissues and organs, and whose signaling activity can be both positively and negatively regulated by endocytosis (reviewed by Yamamoto et al., 2010; Baron, 2012; Schnute et al., 2018; Hosseini-Alghaderi and Baron, 2020). Mutations causing both loss and gain of Notch signaling result in a plethora of different developmental phenotypes (Bray, 2016; Siebel and Lendahl, 2017). Notch is activated by proteolytic removal of its extracellular domain (ECD). This can happen at the cell surface through binding of Notch ligands, which exposes an ADAM10/Kuzbanian S2 cleavage site (Kopan and Ilagan, 2009) or, independently of ligands, at the endosomal/lysosomal surface (Shimizu et al., 2014). For example, in *Drosophila*, the prevention of Notch transfer from the endosome membrane into the ILVs by removal of ESCRT complex activity promotes ligand-independent Notch signaling (Horner et al., 2018). Both ligand-dependent and independent mechanisms lead to the subsequent proteolytic release of the Notch intracellular domain (ICD), allowing it to relocate to the nucleus. There it forms a complex with the transcription factor Suppressor of Hairless (Su[H]) and coactivator Mastermind, which activates transcription (Petcherski and Kimble, 2000; Wilson and Kovall, 2006).

Using *Drosophila*, we identified two means by which Notch can be activated in the endosomal pathway, both of which are ligand-independent and provide a buffer for overall Notch signaling levels (Shimizu et al., 2014). The ring finger domain protein Deltex (Dx) binds to the intracellular domain of Notch and directs full-length Notch trafficking through clathrin-mediated endocytosis (CME) to the late endosome. Dx further acts to prevent Notch transfer into ILVs. Hence Notch is retained on the perimeter endosomal membrane and Notch activation then requires lysosomal fusion. The latter is dependent on the homotypic fusion and protein-sorting (HOPS) complex and TRPML, the lysosomal membrane Ca^2+^ channel (Wilkin et al., 2008; Shimizu et al., 2014). The removal of the Notch extracellular domain is independent of Kuzbanian/ADAM10 and is presumed to occur by exposure to lysosomal proteases. Notch ICD is then released by γ-secretase-dependent S3 cleavage. The second ligand-independent activation mechanism is Kuzbanian/Adam10-dependent and requires early but not late endosomal/lysosomal trafficking components. This class of activation is associated with full-length Notch endocytosis through a clathrin-independent, endocytic pathway (Shimizu et al., 2014). Unlike the ligand-dependent and Dx-driven forms of activation, this class is specifically suppressed by using methyl-beta-cyclodextrin (MβCD)-depletion of cells and by RNAi knockdown of the glycosphingolipid synthesis pathway enzymes such as GLCT-1 (Shimizu et al., 2014). Notch entry into this endocytic route is promoted by the *Drosophila* Nedd4 family protein Suppressor of deltex (Su[dx]), which also binds to the Notch intracellular domain. Human WWP2, a homolog of Su(dx), also binds to human NOTCH3 and suppresses its ectopic activation (Jung et al., 2014). When the HECT ubiquitin ligase domain of *Drosophila* Su(dx) is active then Notch is transferred to the endosomal lumen and signaling is terminated. When the HECT domain is inactive then this transfer does not occur and Notch is retained on the endosome membrane, allowing its activation. With both Dx and Su(dx)-induced Notch endocytosis, full-length Notch localizes to discrete microdomains on the endosome membrane whose nature and composition are unknown (Shimizu et al., 2014). It is not known which of the above mechanisms activates Notch following downregulation of ESCRT function.

Here, we investigated the localization of Notch on the endosome membrane in different activating and downregulatory conditions. We demonstrate differential regulation of Notch, associated with distinct cholesterol-dependent and clathrin-rich membrane microdomains, regulated by ubiquitin ligase activity. We further uncover a novel gate-keeping role for ESCRT complex components in controlling Notch localization to different membrane environments. Despite the prevailing model that ESCRT complexes work together in the process of ILV transfer, we found that there were surprising differences between the mechanisms of Notch activation arising from disruption of ESCRT-I and III components, linked with either Notch retention in cholesterol-rich or transfer to clathrin-rich microdomains, respectively. We further showed that activating mutations of Notch that remove C-terminal regions, similar to mutations found in human cancers and developmental disorders (Weng et al., 2004; Wang et al., 2015; Mašek and Andersson, 2017; Ramain et al., 2001), also shift the activation mechanism toward the late endosomal-dependent activation pathway. Our studies therefore reveal the intimate interplay between Notch endocytic regulators, core endosomal components, and Notch genotype in defining the spatial location in the endosome membrane and the mechanism of Notch signal initiation.

## Results

### Notch trafficking can be directed to discrete cholesterol-rich or clathrin-rich endosome membrane microdomains

**Figure 1.**
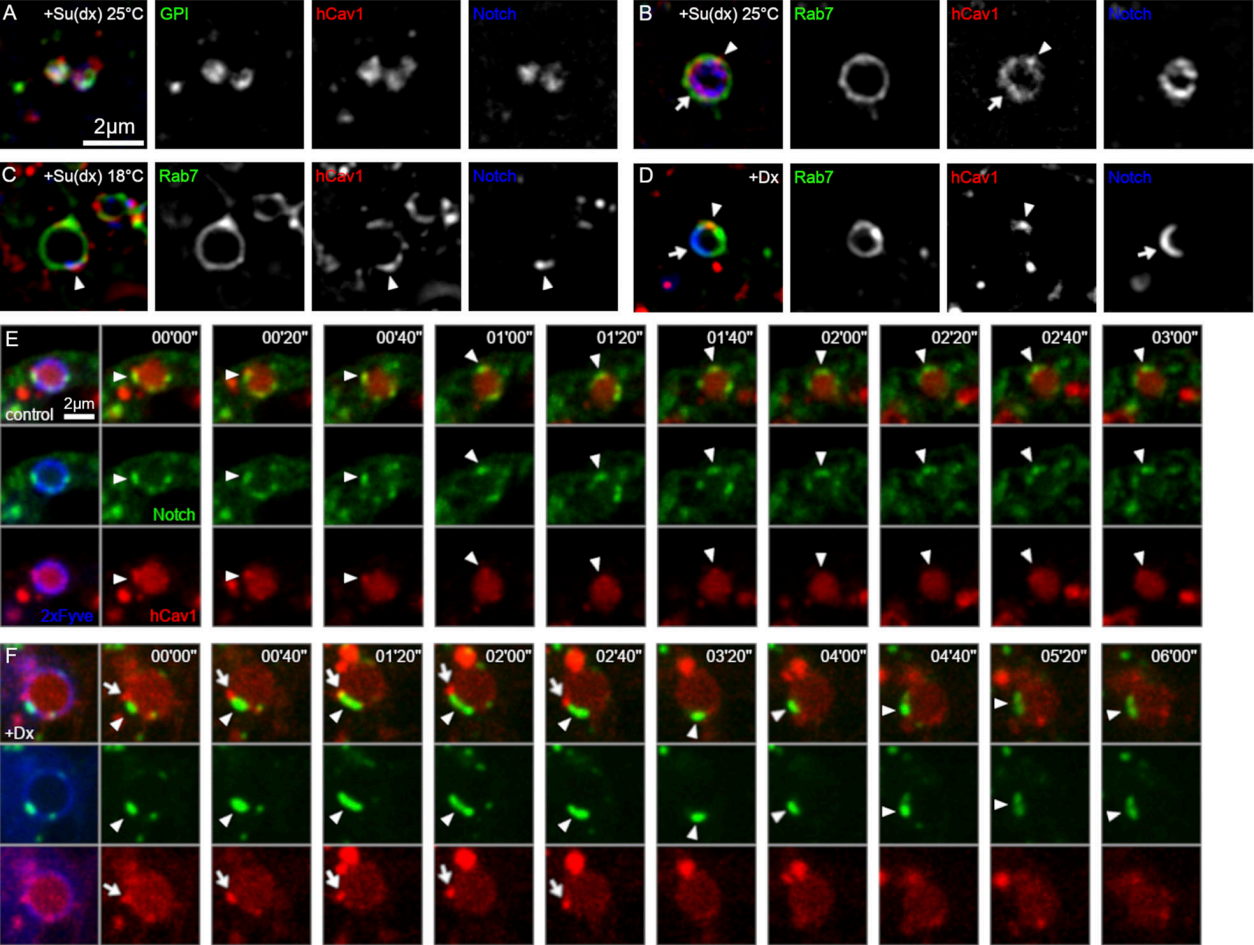
**Notch localization compared with lipid raft-like microdomains on the endosome surface in S2 cells. (A–C)** Colocalization of lipid rafts markers and Notch endocytosed for 60 min at 25°C in Su(dx)-overexpressing S2 cells. **(A)** EGFP-GPI (green), human Cav1-mRFP (red), and internalized Notch^ECD^ antibody (blue). **(B)** EYFP-Rab7 (green), Cav1-mRFP (red), and Notch^ECD^ antibody (blue) at 25°C. Note that Cav1 is mainly found inside the endosome (arrow), but there is also a spot on the limiting membrane (arrowhead). **(C)** EYFP-Rab7 (green), Cav1-mRFP (red), and Notch^ECD^ antibody (blue) at 18°C. Arrowhead marks Notch colocalization with Cav1. **(D)** EYFP-Rab7 (green), Cav1-mRFP (red), and Notch^ECD^ antibody (blue) endocytosed for 60 min at 25°C in Dx-overexpressing S2 cells. Notch (arrow) and Cav1 (arrowhead) are separated into different microdomains on the endosomal surface. Note that without Dx or Su(dx) expression, we were unable to image Notch the limiting endosomal membrane in S2 cells because of the constitutive transfer of Notch into endosomal lumen and more limited endocytic uptake (Shimizu et al., 2014). **(E and F)** Time-lapse images of Notch-EGFP (green), Cav1-mRFP (red), and SNAP-2xFYVE (blue), expressed in control (E), and Dx-expressing (F) S2R+ cells. **(E)** Notch is localized to and moves with Cav1-positive (raft-type) membrane domain on endosomal surface (arrowheads) in control cells. **(F)** In Dx-expressing cells, Notch localizes to discrete patches on the endosome membrane (arrowheads), which are separate from the Cav1-positive membrane domain (arrows).

**Figure S1.**
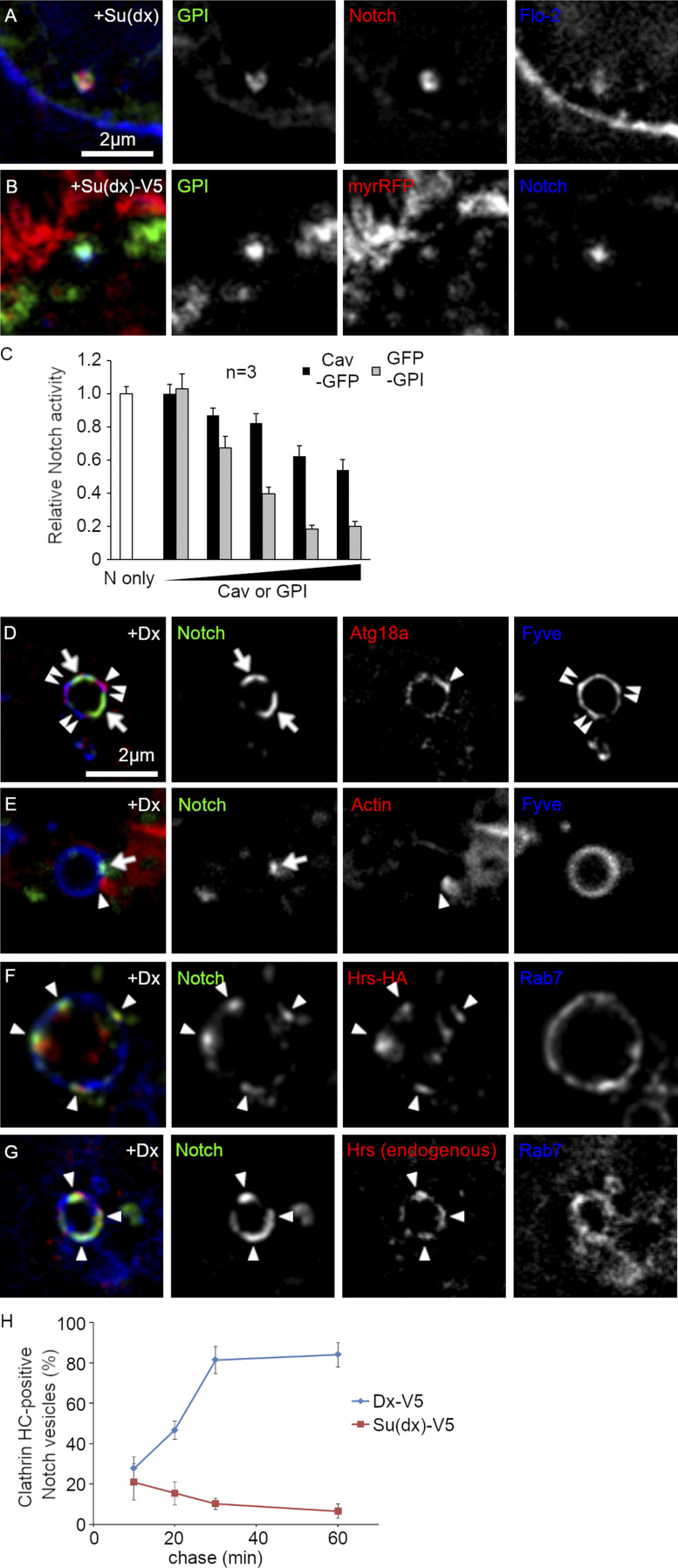
**Microdomain markers in S2 cells. (A and B)** Colocalization of lipid raft markers and Notch endocytosed for 60 min at 25°C in Su(dx) (A) or Su(dx)-V5 (B) overexpressing S2 cells. **(A)** GFP-GPI (green), Notch^ECD^ (red) antibody, and Flotillin-2-HA (blue). **(B)** GFP-GPI (green), myr-RFP (red), and Notch^ECD^ (blue) antibody. **(C)** Luciferase assay of Notch signal activation in S2 cells cotransfected with 0, 0.1, 0.3, 1.0, 3.0, or 10 ng pMT-Cav-GFP or pMT-GFP-GPI. GFP-GPI has a greater impact on Notch signal than Cav-GFP expression. Notch-only represents control Notch-expressing cells that were not cotransfected with Dx or Su(dx). **(D and E)** Localization of Notch^ECD^ antibody endocytosed in S2 cells for 60 min (green) when coexpressed with Dx, on Fyve-positive endosomes compared to Atg18a (D) and Actin (E). **(F and G)** Colocalization of Notch^ECD^ antibody endocytosed for 60 min with Hrs on Rab7-labeled endosomal membrane in S2 cells overexpressing Dx. Overexpressed Hrs-HA (F) or endogenous Hrs (G) show colocalization with Notch on EYFP-Rab7 or mTagBFP2-Rab7-positive late endosomes, respectively. **(H)** Time course of colocalization between Notch^ECD^ antibody endocytosis and mRFP-clathrin heavy chain in Dx or Su(dx)-V5 overexpressing cells. Notch and clathrin colocalize only at a later stage of endocytosis in Dx-expressing cells. Error bars represent SEM. Data from three repeats with 50–100 puncta scored per repeat.

**Figure 2.**
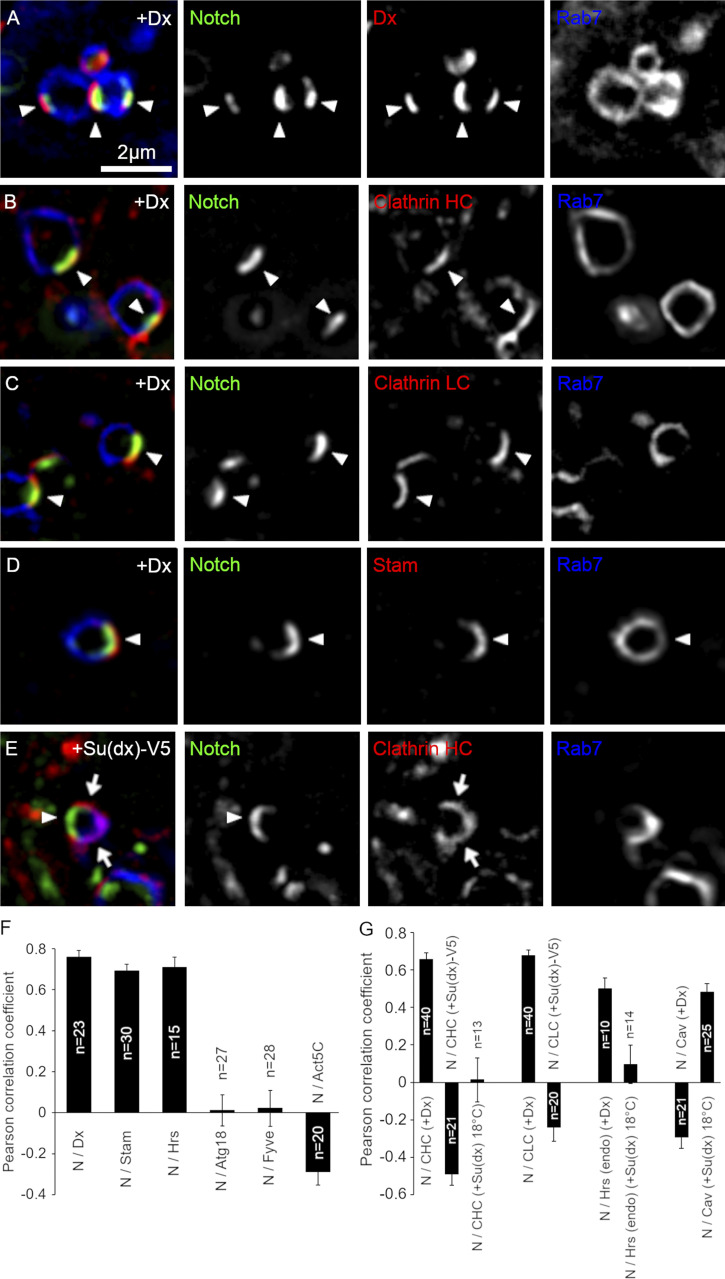
**Deltex-induced colocalization of Notch and ESCRT-0 complex on a microdomain of the endosomal limiting membrane. (A–D)** Localization of Notch^ECD^ antibody endocytosed for 60 min (green) in S2 cells expressing Venus-Dx compared with (A) Venus-Dx (red) and mRFP-Rab7 (blue), (B) EGFP-clathrin heavy chain (red) and mRFP-Rab7 (blue), (C) mRFP-clathrin light chain (red) and EYFP-Rab7 (blue), (D) expressed STAM-HA (red) and EYFP-Rab7 (blue). Arrowheads mark sites of colocalization. **(E)** In S2 cells overexpressing Su(dx)-V5 and mRFP-clathrin heavy chain, Notch (arrowhead) localizes to a discrete patch outside of the clathrin-marked subdomain (arrows). **(F and G)** Fluorescence intensity of Cav1 or CLC (green) and Notch (red) were measured around the limiting membrane of each endosome and Pearson’s correlation coefficient of colocalization between internalized Notch and markers for endosomal microdomains determined, including Stam-HA, Hrs-HA, Atg18-HA, GFP-myc-2xFYVE, in Dx-expressing S2 cells and GFP-Actin5C in Dx-expressing S2-R+ cells (F), and mRFP-clathrin heavy chain, EGFP-clathrin light chain, endogenous Hrs, and mRFP-Caveolin in Dx- or Su(dx)-expressing S2 cells (G). Error bars, SEM, no. of samples indicated in the figure.

Ligand-independent Notch signaling can occur by ADAM10-dependent and independent mechanisms, the latter promoted by the interaction of Notch with Dx (Shimizu et al., 2014). To investigate how different signal activation mechanisms relate to the subdivision of endosomal membrane environments in *Drosophila* S2 cells, we first investigated the localization of Notch compared with markers that bind to distinct lipid-raft-like membrane compositions. Caveolin binds to cholesterol-rich membrane domains (Murata et al., 1995). Although insects do not have caveolae and do not have a homolog of the caveolin gene, we investigated if the expression of human caveolin-1-mRFP (Cav1-mRFP) could be used as a reporter construct for labeling cholesterol-rich microdomains on the endosomes. We previously found that Su(dx) expression increases Notch endocytic uptake in S2 cells compared with the low rates observed in the basal Notch-only transfected condition, and Notch became localized in endosomal compartments marked by EGFP-GPI (Shimizu et al., 2014). We found that EGFP-GPI and Cav1-mRFP localization both coincided with Notch after Su(dx)-promoted endocytosis (Fig. 1 A). Similarly, Notch in EGFP-GPI endosome compartments also colocalized with *Drosophila* Flotillin, another marker of cholesterol-rich membranes, and partly with myristoylated-mRFP (Fig. S1, A and B). Cav1-mRFP was less perturbing for basal Notch signaling (i.e., without coexpression with Dx or Su[dx]) in S2 cells at the expression levels used in this study compared with EGFP-GPI (Fig. S1 C) and had less background staining than other markers. When Notch endocytosis was promoted by Su(dx) coexpression in 25°C culture (ubiquitin ligase active), endocytosed Notch, labeled with antibody uptake, colocalized with Cav1-mRFP, predominantly in the lumen of Rab7-EGFP positive late endosomes (Fig. 1 B). In 18°C culture, when the Su(dx) ubiquitin ligase domain is inactive (Shimizu et al., 2014), Notch was localized to patches of Cav1-mRFP positive membrane on the endosome surface (Fig. 1 C and Fig. 2 G). In contrast, when Notch endocytosis was promoted by Dx coexpression, then Notch was localized in a separate microdomain to the Cav1-mRFP positive region (Fig. 1 D and Fig. 2 G). Note, that we were unable to image Notch on the endosomal limiting membrane in the basal condition (lacking Su[dx] and Dx) when using antibody-uptake experiments due to lower endocytic uptake rates and constitutive transfer into the endosomal lumen (Shimizu et al., 2014).

The different localizations of Notch that occur with Dx and Su(dx) were not artifacts of fixation conditions as the differences were also observed in live cells (Fig. 1, E and F). To monitor Notch localization, we utilized a Notch construct with an EGFP tag inserted in the intracellular domain, C-terminal to the ankyrin repeat region. To mark the endosome membrane, we used a SNAP-tagged 2xFYVE construct with a Far-Red Fluorophore. The latter contains the FYVE domain from Hrs and binds to phosphatidylinositol 3-phosphate and acts as a reporter construct for labeling endosomal membranes (Burd and Emr, 1998). Unlike with the antibody uptake experiment, when observing total Notch expressed in S2R+ cells, without Dx or Su(dx) coexpression, we were able to detect some Notch on limiting membranes. In this basal condition, Notch remained localized to Cav1-mRFP marked microdomains over a time course of several minutes and moved with Cav1-mRFP patches around the endosomal surface (Fig. 1 E and Video 1), consistent with a low level of basal activation in this condition (Fig. S1 C). In contrast, when Dx was coexpressed in live cells, Notch localized separately from Cav1-mRFP marked membrane regions (Fig. 1 F and Video 2).

**Video 1.** **Time-lapse live cell spinning disc confocal imaging of transfected N-EGFP (green) and Cav1-mRFP (red) in control S2-R+ cells, showing synchronized localization and dynamics of both puncta on the endosomal limiting membrane.** Representative frames of this movie are shown in Fig. 1 E. Scale bar: 2 μm. Images captured (EGFP: 100 ms, mRFP: 100 ms, SNAP-Cell 647-SiR: 50 ms) every 10 s over 2 μm at 0.34 µm Z-intervals (seven planes) for 10 min at 25°C, average projection of three planes.

**Video 2.** **Time-lapse live cell spinning disc confocal imaging of transfected N-EGFP (green) and Cav1-mRFP (red) in Dx-expressing S2R+ cells, showing independent localization and dynamics of both puncta on endosomal limiting membrane.** Representative frames of this movie are shown in Fig. 1 F. Scale bar: 2 μm. Images captured (EGFP: 100 ms, mRFP: 100 ms, SNAP-Cell 647-SiR: 50 ms) every 10 s over 2 μm at 0.34 µm Z-intervals (7 planes) for 10 min at 25°C, average projection of three planes.

When Notch was recruited to endosomes by Dx expression, we found that both proteins localized together (Fig. 2, A and F). To further characterize this membrane microdomain, we investigated a number of additional markers. Phosphatidyl-inositol derivatives are known to form subdomains on cellular membranes, and PI3P and PI(3,5)P2 are enriched in early and late endosomes, respectively. Indeed, both FYVE-EGFP (PI3P binding domain) and Atg18a (PI(3,5)P2 binding protein) staining showed uniform, ring-like distribution on the endosomal surface with some punctate enrichment, but these hot spots did not colocalize with the Dx-domain (Fig. 2 F and Fig. S1 D). Rab7 also has ring-like localization; however, interestingly, the staining is partially excluded from the Dx domain (Fig. 2, A–D). Actin filaments are also known to form clusters on specific sites of the endosomal surface such as the WASH domain and actin-comet (Taunton et al., 2000). In Dx/Notch expressing S2-R+ cells, Actin-EGFP formed a tail-like structure on endosomes, but we did not find any correlation between any of these structures with the Dx domain (Fig. 2 F and Fig. S1 E). Clathrin has previously been shown to mark subregions of endosomal membranes as a flat clathrin coat (Raiborg et al., 2006). When Notch endocytosis was promoted by Dx, Notch colocalized with clathrin heavy and light chains, shown in Fig. 2. B, C, and G. The ESCRT-0 components Hrs and STAM have previously been found to localize to clathrin-enriched endosome microdomains in Rab7-positive endosomes (Raiborg et al., 2002, 2006), and we found that Notch also localized with expressed STAM and Hrs when either was coexpressed with Dx (Fig. 2, D and F; and Fig. S1 F) and with endogenous Hrs (Fig. 2 G and Fig. S1 G). However, when Notch endocytosis was promoted by the expression of Su(dx)-V5 (a C-terminally tagged Su[dx] construct, which is ubiquitin ligase defective) or Su(dx) at 18°C, then Notch localized on the endosome membrane in a location distinct from the clathrin-enriched region (Fig. 2, E and G).

**Figure 3.**
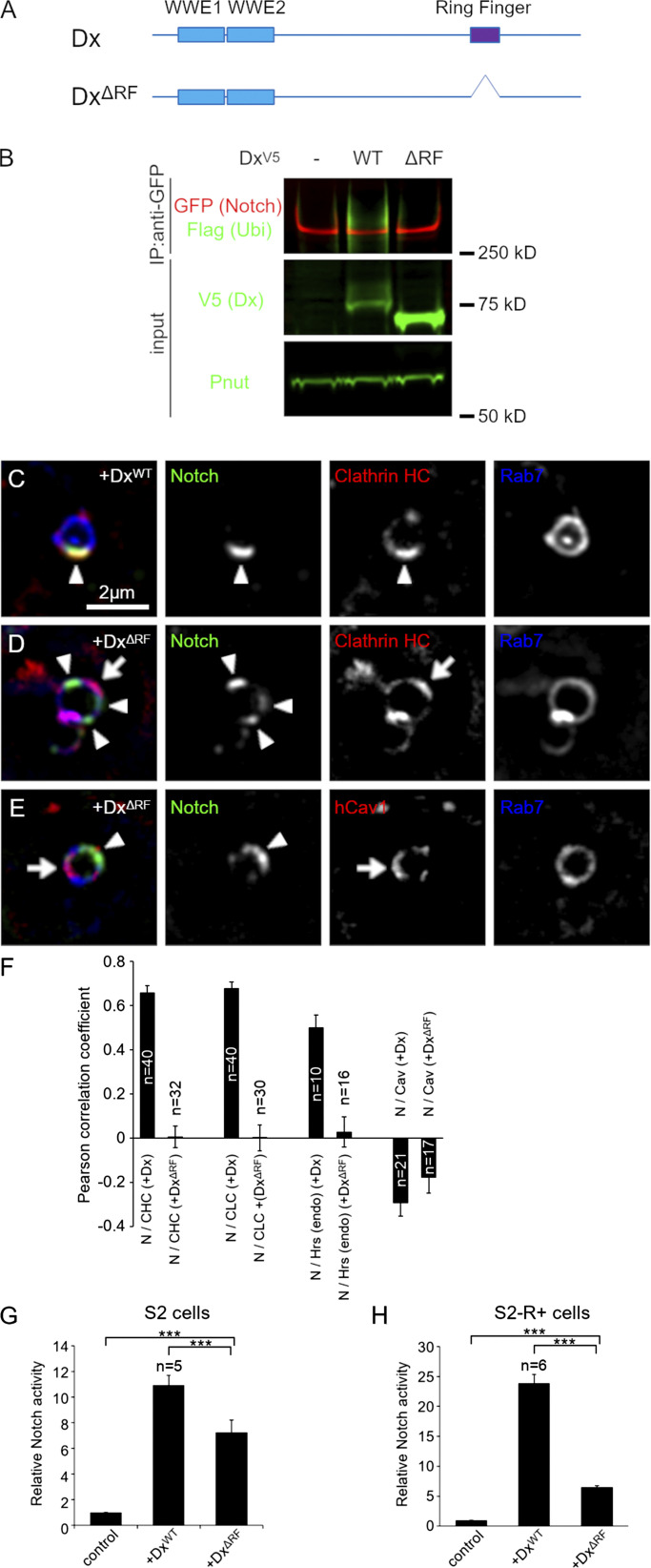
**Regulatory role of Deltex-mediated ubiquitination in Notch recruitment to the clathrin-positive endosomal subdomain. (A)** Schematic diagram of *Drosophila* Dx protein and ΔRF (ring finger-truncated). **(B)** Ring finger-dependent Notch ubiquitination by Dx in S2 cells. EGFP-tagged Notch, Flag-ubiquitin, and Dx-V5 (WT or ΔRF) were overexpressed in S2 cells and Notch was pulled down by GFP-trap. Notch ubiquitination was detected by the M2 Flag antibody. **(C–E)** Localization of Notch^ECD^ antibody endocytosed for 60 min (green) in S2 cells expressing EYFP-Rab7 (blue) and (C) wild-type Dx (Dx^WT^) and mRFP-clathrin heavy chain (red), (D) Dx^ΔRF^ and mRFP-clathrin heavy chain (red), and (E) Dx^ΔRF^ and mRFP-Cav1 (red). Notch (arrowheads) failed to colocalize with clathrin (arrow in D) and with caveolin (arrow in E) in Dx^ΔRF^-expressing cells. Note that the endosomal clathrin domain exists without the ring finger domain of Dx. **(F)** Pearson’s correlation coefficient of colocalization between internalized Notch and markers for endosomal domains, mRFP-clathrin heavy chain, EGFP-clathrin light chain, endogenous Hrs, and mRFP-Caveolin in WT Dx- or DxΔRF-expressing S2 cells. **(G and H)** Functional analysis of Dx Ring finger domain in Notch signal activation by luciferase assay in S2 cells (G) and in S2-R+ cells (H). *** indicates P < 0.001 by two tailed *t* test. Error bars are SEM, sample sizes are indicated on the figure. Source data are available for this figure: SourceData F3.

ESCRT-0 proteins have ubiquitin-binding domains that are able to recruit ubiquitinated cargo. Dx promotes Notch ubiquitination (Hori et al., 2011) and we wondered if this was required for Notch recruitment to the clathrin-rich membrane region. When we expressed a Dx construct lacking the ring finger ubiquitin ligase domain (Fig. 3 A), then Notch was not ubiquitinated (Fig. 3 B). Interestingly, the ring finger domain was not required for Dx to promote Notch endocytosis; however, endocytosed Notch occupied a different discrete membrane region that did not overlap with caveolin, clathrin, or endogenous Hrs markers (Fig. 3, C–F). This change of localization was associated with reduced signaling in both S2 and S2-R+ cell lines compared with full-length Dx (Fig. 3, G and H), indicating that the precise localization within membrane microdomains is crucial for forming the optimum platform for efficient ligand-independent Notch activation. We next investigated, in *Drosophila* S2 cells, the time course of Notch localization with clathrin heavy chain when Dx is expressed, using pulse-chase endocytic uptake assay of Notch. We found that Notch became progressively colocalized with clathrin over a 1-h time course, reaching a plateau of clathrin colocalization after around 30 min (Fig. S1 H). When Notch endocytosis was stimulated by expression of Su(dx)-V5, then Notch colocalization with clathrin remained low throughout the time course of the experiment (Fig. S1 H).

**Figure 4.**
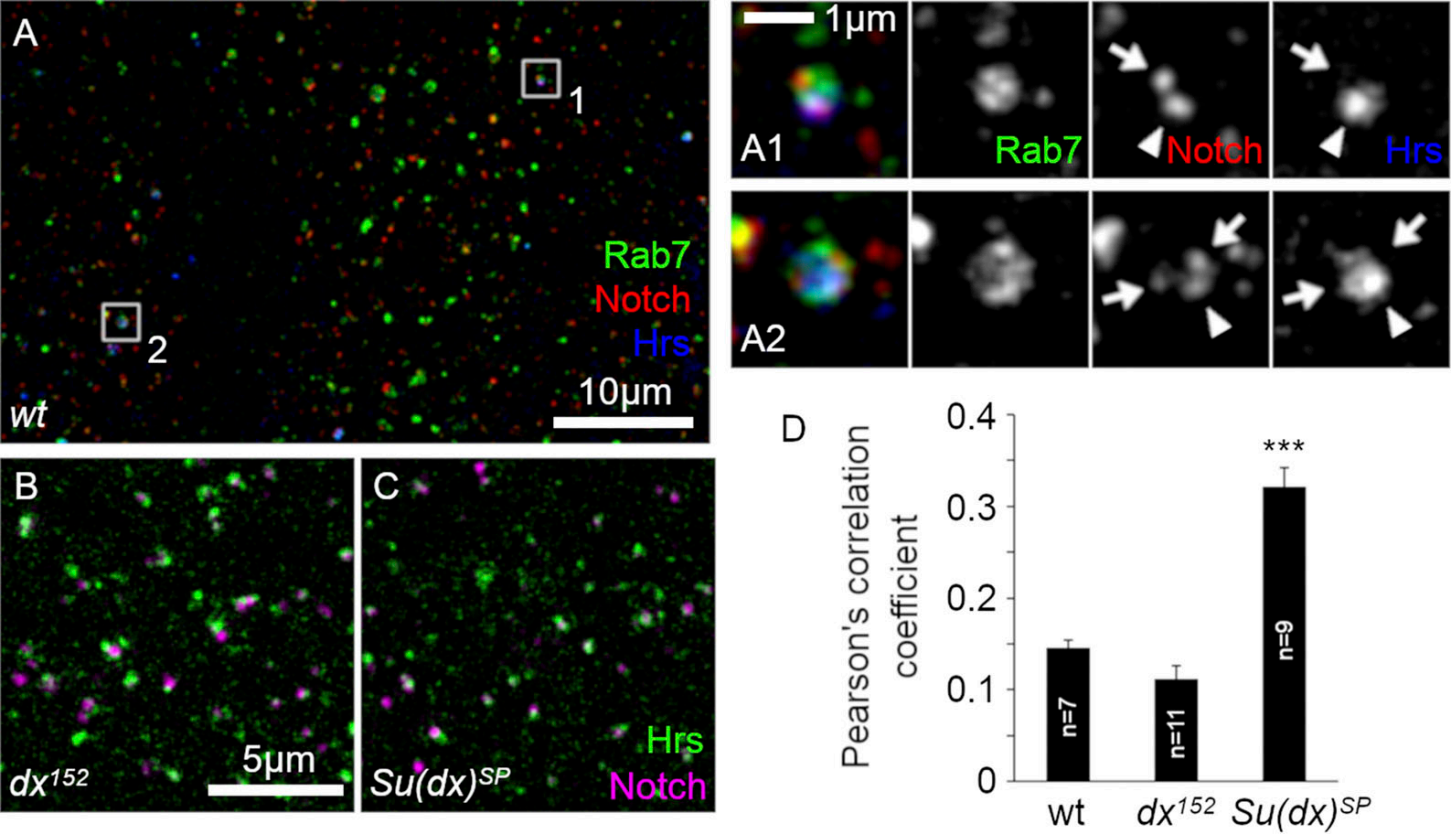
**Notch localization to the Dx/ESCRT-0-domain in vivo. (A)** Localization of endogenous Rab7 (green), Notch (red), and Hrs (blue) in a third-instar wing disc. **(A1 and A2)** Zoom and merge of Rab7 (green), Notch (red), and Hrs (blue). Zoomed-in images of endosomes (white boxes in A) showing Notch localization on Hrs positive (arrowheads) and negative (arrows) microdomains on the endosomal surface. **(B–D)** Distribution of endogenous Notch (purple) and Hrs (green) in wing discs. Notch and Hrs are separated in *dx*^*152*^ mutant (B) but colocalized in *Su(dx)*^*SP*^ mutant disc (C). **(D)** Mean Pearson’s correlation coefficient values of Notch and Hrs in yw, dx^152^, and Su(dx)^SP^. Error bars are SEM, ***P < 0.001 by two tailed *t* test, sample sizes are indicated in the figure.

**Figure S2.**
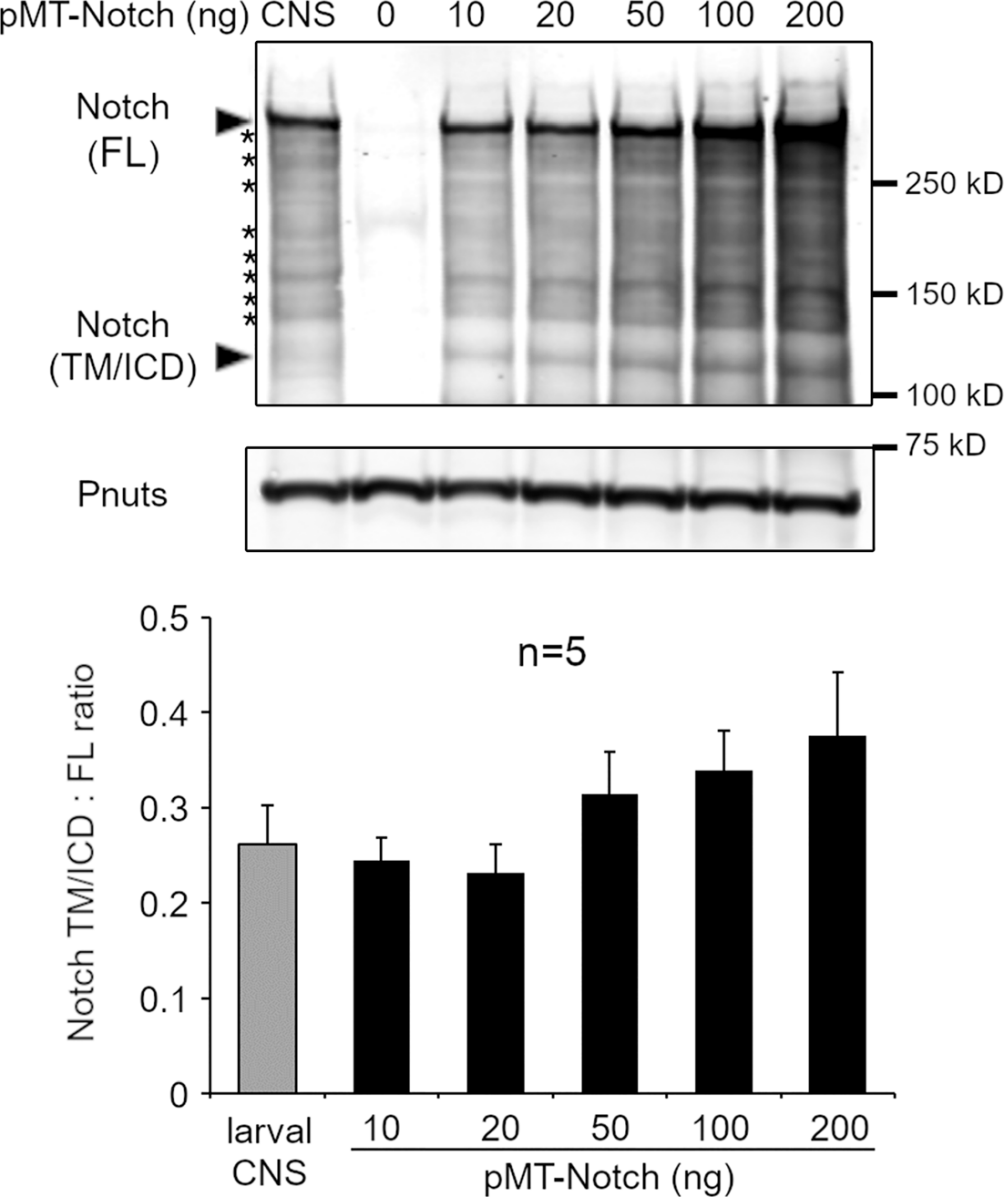
**Comparison of expression levels and processing between transfected cells and endogenous Notch from dissected fly tissue.** Western blotting to show the effect of Notch overexpression on Notch processing in S2 cells and quantification of the ratio of Notch fragments lacking ECD, i.e., transmembrane (TM) tethered/soluble ICD domains, located around 120 kD, to full-length protein around 300 kD. The graph below shows quantification from five repeats showing no change in ratio, up to a 20 ng limit used in this study. Characteristic fragmentation pattern of Notch (asterisks) is in common between endogenous Notch from tissue extracts and Notch expressed in S2 cells. Source data are available for this figure: SourceData FS2.

We considered whether Notch introduced into S2 cells by transient transfection may behave differently to endogenous Notch in vivo due to its overexpression. In vivo, in *Drosophila* wing imaginal disc cells, endogenous Notch in Rab7-positive endosomes was also distributed in differently marked endosomal microdomains, marked by the presence/or absence of Hrs (Fig. 4 A). Su(dx) mutation resulted in a strong shift toward increased colocalization of Notch with Hrs, while mutation of dx resulted in a smaller shift toward less colocalization with Hrs (Fig. 4, B–D). We examined, by Western blot, cell extracts derived from the larval central nervous system (CNS) and compared them with S2 cell extracts following transient transfection with varying amounts of the pMT-Notch expression vector (Fig. S2). The results showed that endogenous and transfected Notch showed a similar, characteristic fragmentation pattern, indicating equivalent processing of Notch both in vivo and in cell culture. The ratio of processed Notch forms that lack ECD to full-length (FL) Notch was found to be identical up to 20 ng of pMT-Notch, which is the upper limit used in this study and the overall expression levels were similar to endogenous Notch.

These results, therefore, indicate that Notch becomes localized to different membrane microdomains on the endosomal surface that are marked by distinct membrane and protein compositions, and these localizations are under the control of Notch interacting ubiquitin ligase proteins. Precise localization determines the efficiency of Notch ligand-independent activation.

### Different ESCRT complexes suppress distinct activation mechanisms of Notch signaling

**Figure 5.**
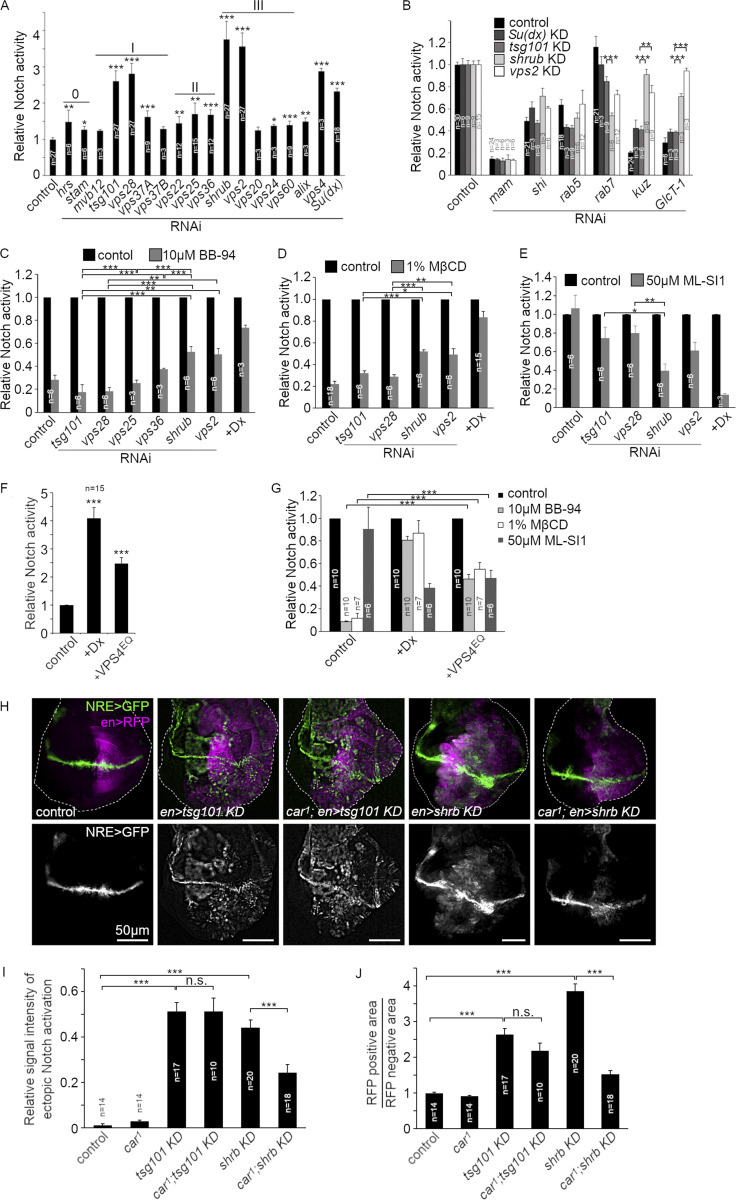
**Different mechanisms of Notch signal activation induced by ESCRT-I, II, and III-Knockdown. (A)** Fold activation of Notch signal in S2 cells by NRE-firefly luciferase assay after RNAi knockdown of ESCRT complexes and Su(dx). **(B)** Notch signaling after double knockdown of ESCRT components combined with mastermind, shibire, Rab5, Rab7, Kuzbanian, and GlcT-1 RNAi. **(C–E)** ESCRT KD-induced Notch signal activity after cells treated with (C) 10 μM BB-94, metalloprotease inhibitor, (D) cholesterol-depletion by 1% MβCD, or (E) 50 μM ML-SI1, TRPML inhibitor. **(F)** Notch activation by overexpressing VPS4^EQ^ in S2 cells. **(G)** VPS4^EQ^-induced Notch signal when cells treated with BB-94, MβCD, or ML-SI1. **(H–J)** In vivo analysis of Notch signal in wing discs. **(H)** Ectopic Notch signal activation and tumor-like phenotype induced in the posterior half of wing discs by engrailed-gal4 and UAS-tsg101 RNAi or UAS-shrub RNAi. Shrub KD-induced Notch signal and the overproliferation phenotype can be suppressed by in *car*^*1*^ genetic background but TSG101 KD-induced phenotypes are not affected. **(I)** Fluorescence intensity of NRE-GFP reporter in RFP-positive ventral side of the wing discs, normalized by NRE-GFP intensity along D-V boundary in RFP-negative side. **(J)** The overproliferation phenotype quantified as a ratio between RFP-positive and -negative area in each wing disc. *, **, and *** indicate P < 0.05, 0.01, and 0.001, respectively, by two-tailed Student's *t* test. Error bars are SEM and sample sizes are indicated in the figure.

**Figure S3.**
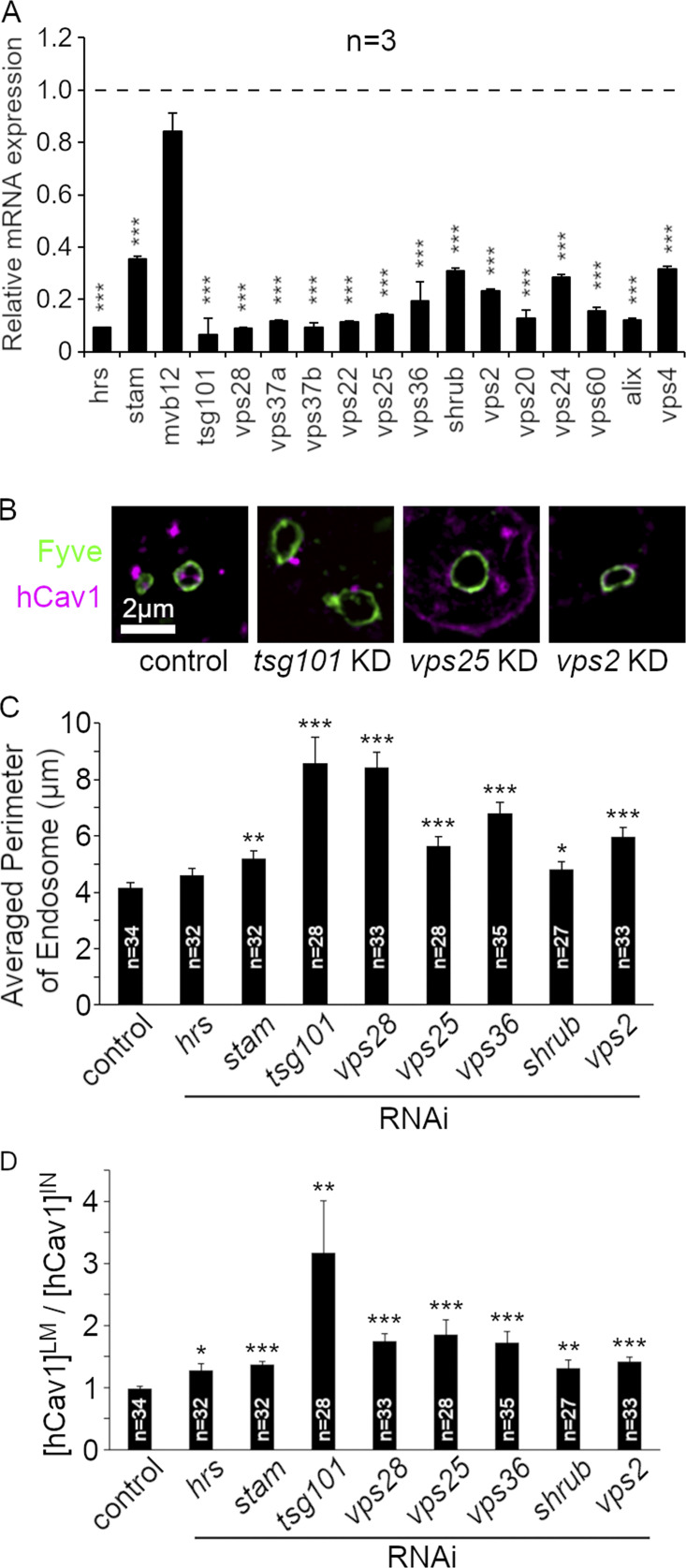
**Consequences of ESCRT knockdown on endosomal perimeter size and Cav-mRFP distribution. (A)** Real-time qPCR analysis of RNAi for ESCRT complexes in S2 cells. The S2 cells were treated with dsRNA for each ESCRT component and mRNA expression of the gene was analyzed by real-time qPCR to compare with the expression in parental S2 cells. **(B)** Images of human Caveolin-1-mRFP distribution on GFP-2xFYVE-positive endosomes. Cav-mRFP localizes to the intraluminal space in control cells but knocking down ESCRT components by RNAi changes Cav-mRFP distribution to the limiting membrane, and enlarges endosomes. **(C)** Mean perimeter size of GFP-2xFYVE-positive endosomes in ESCRT KD cells. **(D)** The ratio of Cav-mRFP intensity on limiting membrane/in intraluminal space of endosome. In A, C, and D, *, **, and *** indicate P < 0.05, 0.01, and 0.001, respectively, compared with control cells, by two-tailed *t*-test. Error bars represent SEM. Sample numbers are indicated in the figure.

To investigate the mechanism by which loss of ESCRT function activates Notch, we examined, in S2 cell culture, the effects of knockdown of a panel of components from ESCRT-0, I, II, and III complexes, and also that of Su(dx) on the basal Notch activity. We found that knockdown of Su(dx) and several ESCRT components significantly increased Notch signaling (Fig. 5 A), notably ESCRT-I components TSG101 and VPS28, ESCRT-III components Shrub, VPS2, and the ESCRT-III regulator VPS4. The efficiency of knockdown was measured by qPCR (Fig. S3 A), and we further confirmed that RNAi knockdown of selected ESCRT components affected endosome ILV formation and membrane perimeter (Fig. S3, B–D). As expected, the knockdown of ESCRT-I to III components resulted in the enlargement of the endosomal perimeter marked with EGFP-FYVE (Fig. S3, B and C), consistent with reduced formation of ILVs. In control endosomes, Cav1-mRFP was located on the endosomal membrane or in the intraluminal vesicles (Fig. S3, B and D). RNAi knockdown of ESCRT-I, II, and III complex components reduced the amount of intraluminal staining of Cav1-mRFP compared with Cav1-mRFP at the limiting membrane (Fig. S3, B and D).

**Figure S4.**
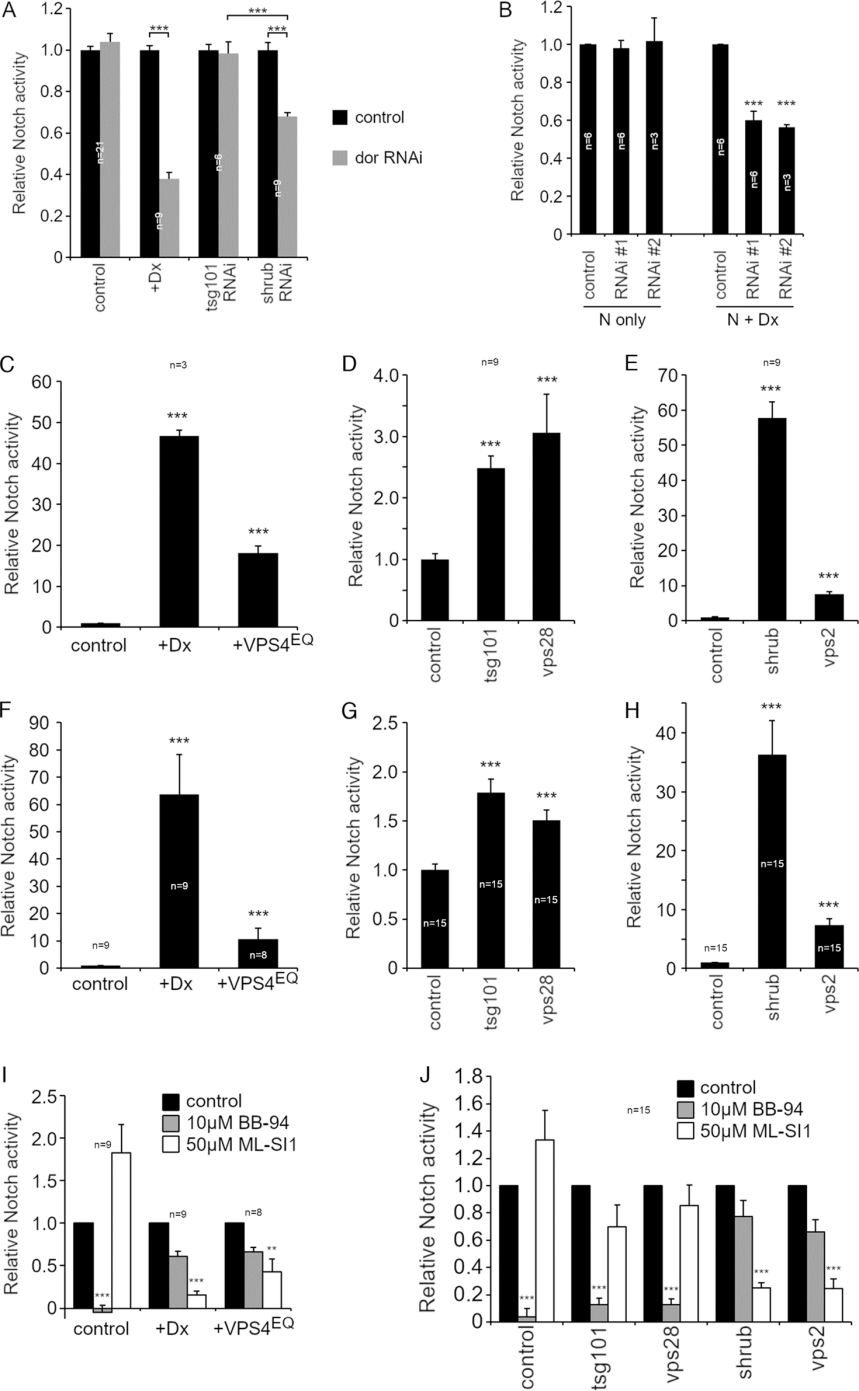
**Discrimination between Notch activation mechanisms in transient transfected and stable cell lines. (A)** Dor RNAi reduces Dx-induced Notch signal, and the Notch signal arising from shrub knockdown, but not the basal Notch signal or that resulting from tsg101 knockdown. **(B)** Two different TRPML RNAi knockdowns reduce Dx-induced Notch signal but not basal Notch activity. Error bars, SEM, *** indicates P < 0.001 by two-tailed *t* test. Sample numbers are indicated in the figure. **(C–J)** Comparison of transiently transfected and S2-R+ stable Notch expressing cell line. Fold activation of Notch signal in S2-R+ cells measured by NRE-firefly luciferase assay with overexpression of Dx and VPS4^EQ^ (C and F) or with RNAi knockdown of ESCRT-I (D and G) or -III components (E and H). S2-R+ cells were transiently expressing Notch (C–E) or the pMT-Notch stable cell line was used (F–H). **(I and J)** Pharmacological analysis of Notch signaling pathways induced by Dx and VPS4^EQ^ (I) or RNAi knockdown of ESCRT complexes (J) in the stable pMT-Notch/S2-R+ cell line, treated with 10 μM BB-94 or 50 μM ML-SI1. ** and *** indicate P < 0.01 and 0.001, respectively by two-tailed *t* test. Error bars represent SEM; number of samples indicated in the figure.

We next investigated the mechanism by which Notch signaling is increased following ESCRT-I and III knockdown. RNAi of Rab5 and Shibire (*Drosophila* Dynamin), which are required for both ligand-independent activation mechanisms (Shimizu et al., 2014) and the transcriptional coactivator Mastermind (Wilson and Kovall, 2006), reduced Notch activity that arose from knockdown of Su(dx) and all of the ESCRT components tested (Fig. 5 B). However, the knockdown of Rab7 revealed differential requirements for signaling. There was little consequence of Rab7 depletion on activity arising from Su(dx) and TSG101 (ESCRT-I) knockdown, an intermediate effect on signaling due to depletion of VPS2 (ESCRT-III), and a stronger effect on the consequences of knockdown of Shrub (ESCRT-III) (Fig. 5 B). HOPS complex function, reduced by deep orange (dor) knockdown, was also required for the increase of Notch signal after Shrub RNAi treatment but not after TSG101 knockdown (Fig. S4 A). Treatment of cells with RNAi targeting ADAM10/Kuzbanian or GLCT-1 RNAi had the reverse outcome. Signaling from Su(dx) and TSG101 knockdown was significantly reduced while signaling from VPS2 and Shrub knockdown was weakly or not affected (Fig. 5 B). The results indicated that Notch signaling arising from the knockdown of different ESCRT complex components in S2 cells is initiated by different mechanisms, either by the basal mechanism (after ESCRT-I KD) or by a mechanism with similar requirements to that arising when Dx is coexpressed (after ESCRT-III KD).

To further investigate the differing Notch signal activation requirements on depletion of different ESCRT components, we investigated the effect of treatment with the metalloprotease inhibitor BB-94 to inhibit ADAM10/Kuzbanian activity. The latter has proven effective in distinguishing Dx-induced and basal Notch signaling mechanisms (Shimizu et al., 2014). Notch signaling that was activated by the depletion of ESCRT-I complex components TSG101 and VPS28 was significantly reduced by BB-94 treatment. However, when signaling was activated by depletion of ESCRT-III components Shrub and VPS2, there was reduced sensitivity to BB-94, similar to when Notch is activated by Dx (Fig. 5 C). Depletion of the ESCRT-II component VPS36 had an intermediate metalloprotease dependency. Therefore, when ESCRT function is blocked at later stages, there is a shift toward an activation mechanism that is independent of metalloprotease. We saw a similar discrimination between different Notch activation conditions when we investigated the consequences of cholesterol stripping of cells with MβCD (Fig. 5 D). Notch signaling by ESCRT-I component depletion was more sensitive to this treatment than when the ESCRT-III complex components were knocked down. Previously, we have shown that overexpression of TRPML, a lysosomal calcium channel involved in endolysomal fusion/fission cycles, greatly enhances Dx-induced signaling (Shimizu et al., 2014). RNAi knockdown of TRPML specifically reduces signaling induced by Dx but not the activity of the basal Notch route (Fig. S4 B). Treatment of cells with TRPML inhibitor ML-SI1 replicated the RNAi results (Fig. 5 E) as basal Notch signaling was unaffected by this treatment, while Dx-induced Notch signaling was strongly affected. Notch signaling arising in conditions of ESCRT-III knockdown showed an intermediate dependency on TRPML activity while signaling after ESCRT-I knockdown was not significantly reduced by ML-SI1 (Fig. 5 E). We confirmed a shift to a Dx-like signaling mode by expressing dominant negative E228Q mutation (Votteler et al., 2016) of human VPS4 (VPS4^EQ^), which increased basal levels of Notch activation in the absence of Dx coexpression (Fig. 5 F). We found that Notch signaling in the presence of VPS4^EQ^ was less dependent on ADAM10 and more dependent on TRPML than basal Notch activation, thus behaving similarly to ESCRT-III knockdown (Fig. 5 G). Similar results, showing discrimination of requirements between activation mechanisms after ESCRT-I or III knockdown, were observed for both transiently transfected and stable (clonal), Notch-expressing S2-R+ cells, showing that the different ligand-independent mechanisms were not derived from variations of transfection levels in a population of transiently transfected cells (Fig. S4, C–J). Interestingly in S2-R+ cells, Dx-induced signaling was more efficiently induced compared with basal Notch signaling, but this was the case for both transiently transfected and stable cell lines.

We confirmed that different Notch activation requirements could also be observed in vivo by expression of RNAi for TSG101 and Shrub in *Drosophila* wing imaginal discs (Fig. 5, H–J). To test for alternative pathways, we disrupted the HOPS complex function using the *carnation*^1^ (*car*^*1*^) mutant. Car, like Dor, contributes to stages of late endosome biogenesis and lysosomal fusion (Sevrioukov et al., 1999; Sriram et al., 2003; Gailite et al., 2012) and, because *car*^*1*^ is a recessive viable hypomorphic missense allele (Sevrioukov et al., 1999), it allowed us to test effects on Notch in wing discs in homozygous mutant flies. We have previously shown *car*^*1*^ to specifically block only the Dx-induced mode of Notch activation in vivo (Wilkin et al., 2008, Yamada et al., 2011). Activation of Notch signaling and overproliferation, resulting from Shrub knockdown, was strongly suppressed by a mutation of the *car*^*1*^. In contrast, Notch signaling and overproliferation that arose from TSG101 knockdown were unaffected by this mutant background (Fig. 5, H–J). These results indicate that different ESCRT components act in vitro and in vivo to suppress ectopic Notch activation that arises by distinct mechanisms.

### Distinct outcomes of different ESCRT component knockdowns on Notch localization in endosomal membrane microdomains

**Figure 6.**
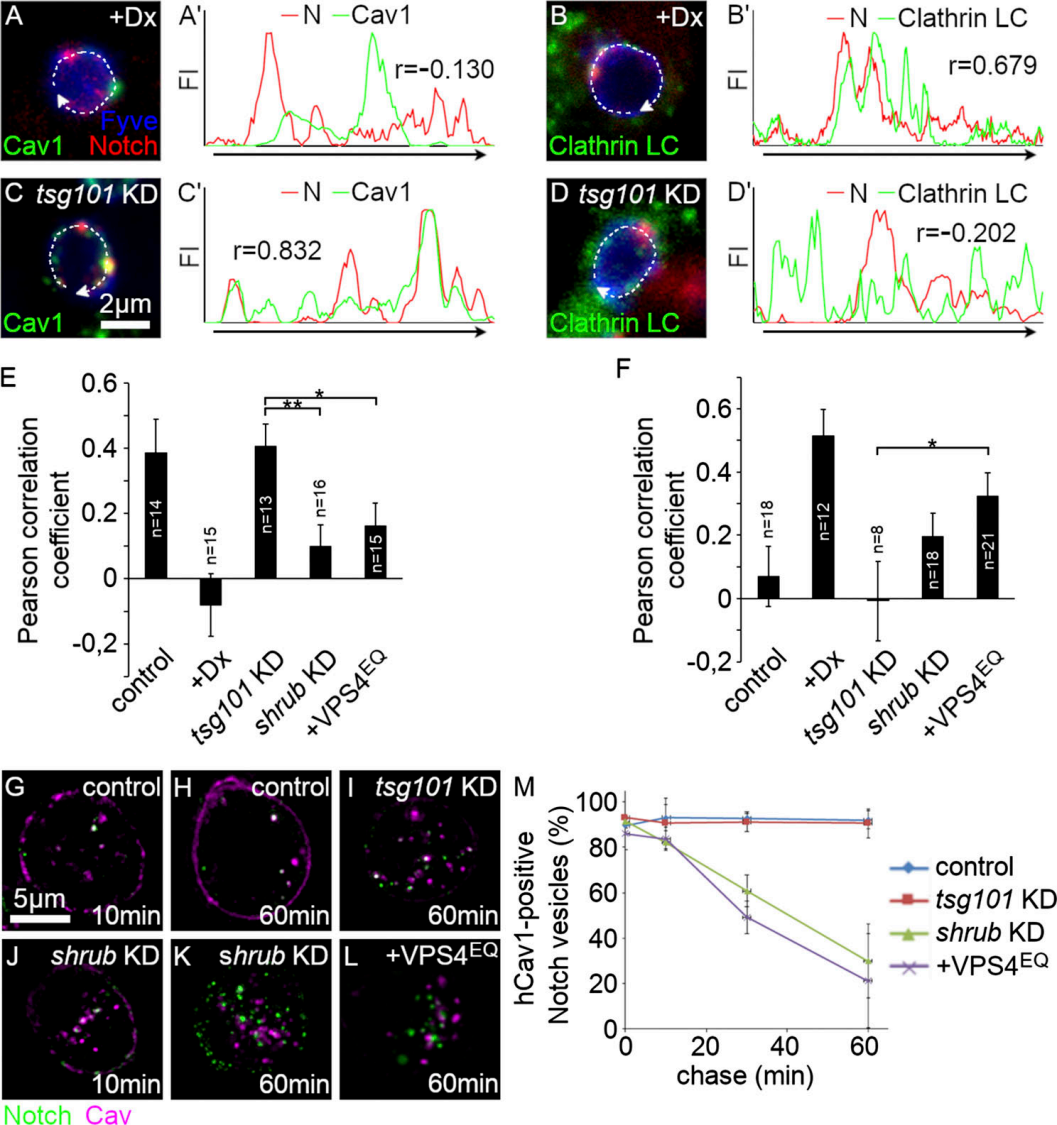
**Change in Notch distribution on the endosomal limiting membrane after ESCRT-III knock-down. (A–D)** Microdomains of the limiting membrane were labeled with Cav1-EGFP (green in A and C) or EGFP-clathrin light chain (green in B and D) together with Notch^ECD^ (red), and mCherry-2xFYVE, an endosomal membrane marker (blue), expressed in S2R+ cells. **(A′–D′)** Fluorescence intensity of Cav1 or CLC (green) and Notch (red) were measured and plotted along the limiting membrane of each endosome (curved arrows in A–D). **(E and F)** Quantification of Pearson’s correlation coefficient. **(E)** Notch/Cav1 colocalization in control S2R+ and Tsg101 KD cells and reduced (or no) correlation in Shrub KD and Dx/VPS4^EQ^ expressing cells. **(F)** Independent distribution of Notch and clathrin light chain in control S2R+ and Tsg101 KD cells and correlative localization in Shrub KD and Dx/VPS4^EQ^ expressing cells. **(G–M)** Downregulation of ESCRT-III function alters Notch localization on the endosome membrane. **(G–L)** Images of Notch^ECD^ antibody (green) endocytosis-uptake assay at indicated chase times in control (G and H), Tsg101 KD (I), Shrub KD (J and K), and VPS4^EQ^ expressing (L) S2 cells, Cav1-mRFP (purple). **(M)** Time course of colocalization between Notch^ECD^ antibody uptake and Cav-1-mRFP showing percentage of Notch^ECD^ on Cav1-mRFP positive spots, scored in control, Tsg101 KD, Shrub KD, and VPS4^EQ^ overexpressing cells. Error bars in E, F, and M are SEM. In E and F, sample sizes are indicated in the figure. *, ** indicate P < 0.05 and 0.01, respectively, by two-tailed Student’s *t* test. In M, data is from three experimental repeats with 50–100 puncta scored per repeat; error bars represent SEM.

To investigate whether the depletion of different ESCRT complex components is associated with different Notch localizations on the endosomal membrane, we used RNAi to knockdown ESCRT components in *Drosophila* S2R+ cells. We examined the localization of Notch on FYVE-positive endosomes compared with Cav1-EGFP-labeled endosome membrane microdomains. We found that when cells were treated with TSG101 RNAi (ESCRT-I complex component), endocytosed Notch colocalized to Cav1-EGFP membrane microdomains, with the proportion of colocalization similar to basal endocytosis conditions, i.e., cells expressing Notch without Dx or Su(dx) (Fig. 6, C and E). However, when the ESCRT-III complex component Shrub was knocked down or when VPS4^EQ^ was expressed, then a significant proportion of endocytosed Notch relocalized to endosome membrane locations that did not colocalize with Cav1-EGFP (Fig. 6 E). This distribution was similar to the localization of Notch when Dx was coexpressed (Fig. 6, A and E).

We performed a similar experiment to investigate Notch localization compared with EGFP-clathrin light chain (Fig. 6, B, D, and F). We found that treatment of cells with TSG101 RNAi produced no colocalization of endocytosed Notch with clathrin, similar to basal conditions when Notch is expressed in S2 cells without Dx (Fig. 6, D and F). In contrast, treatment of cells with Shrub RNAi or coexpression of VPS4^EQ^ resulted in a shift of Notch into clathrin-positive locations (Fig. 6 F), although less than when Dx is expressed (Fig. 6, B and F), consistent with the intermediate requirement for components that are normally associated with the Dx pathway.

To address the time dependency of Notch relocation out of the Cav1-mRFP positive endosomal membrane domains, we examined the proportion of endosome-localized Notch in Cav1-mRFP marked endosome membrane domains over a 60-min endocytic uptake time course (Fig. 6, G–M). When cells were treated with RNAi targeting TSG101, Notch colocalized with Cav1-mRFP throughout the time course, similar to Notch localization in basal uptake conditions (Fig. 6, G–I and M). When cells were treated with RNAi targeting Shrub or when Notch was coexpressed with VPS4^EQ^, then Notch was initially localized to Cav1-mRFP positive domains, but over the 1-h time course, there was a progressive decrease in the proportion of Notch colocalized with Cav1-mRFP (Fig. 6, J–M).

**Figure 7.**
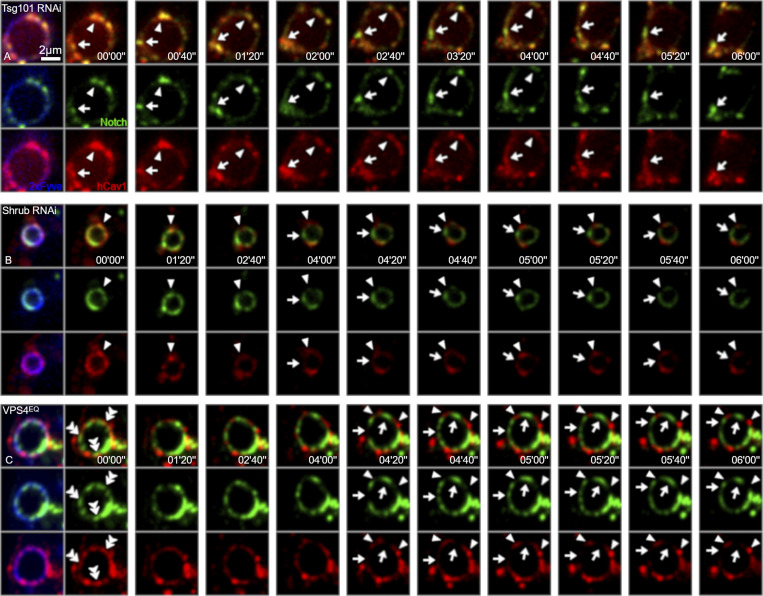
**ESCRT-III dysfunction-induced lateral transition of Notch between membrane microdomains. (A–C)** Time-lapse live cell imaging of Notch-EGFP (green), Cav1-mRFP (red), and SNAP-2xFYVE (blue) in Tsg101 KD (A), Shrub KD (B), and VPS4^EQ^ overexpressing (C) S2R+ cells. **(A)** Individual Notch clusters remain within Cav1-positive domains on the limiting membrane (arrows and arrowheads). **(B)** In Shrub KD cells, Notch is initially found overlapping Cav1 positive (raft-type) membrane domain, but after 04′00″, Notch (arrows), and Cav1 (arrowheads) are separated into different microdomains of the endosome surface. **(C)** Although Notch clusters and the raft-like domains partially colocalize on the endosomal surface at time 00′00″, Notch (arrows) is gradually excluded from the Cav1 domains (arrowheads) and is sorted into distinguishable microdomains at 06′00″.

**Figure S5.**
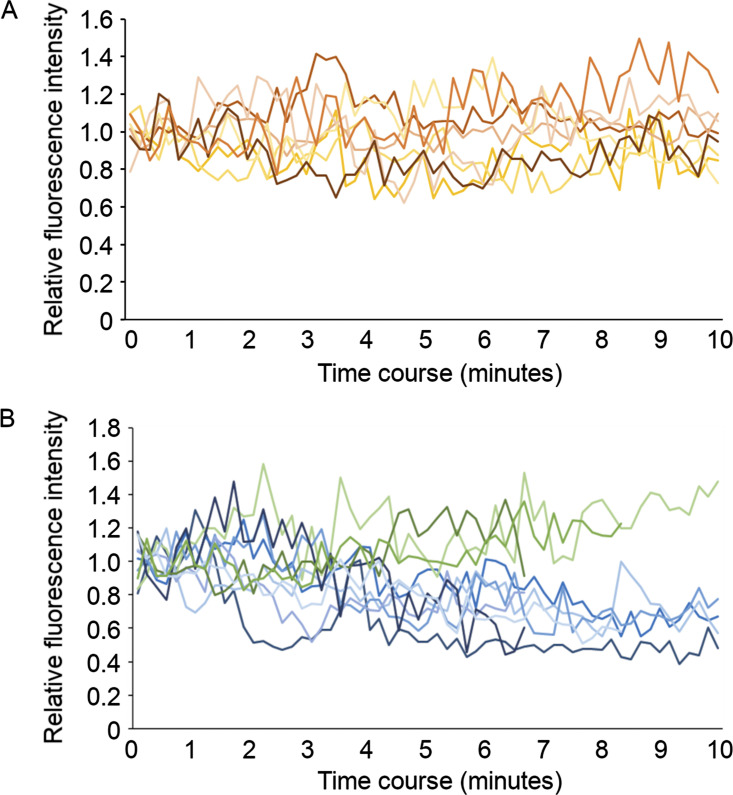
**Transition of Notch localization from raft-type membrane microdomain to non-raft domain.** Cav-mRFP fluorescence intensity on each Notch-GFP spot was normalized by average intensity at the first five time points (from 0 to 40 s). **(A and B)** Notch-GFP remains within Caveolin-positive membrane microdomain over 10 min in Tsg101 KD cells (A), whereas the Cav-mRFP fluorescence declines in majority of Notch-GFP spots in Shrub KD cells (B).

The progressive shift in localization out of Cav1-mRFP positive locations suggests that Notch is initially endocytosed into the Cav1-mRFP endosome membrane domain but, in cells with disrupted ESCRT-III, there is a shift in Notch localization out of this location. An alternative explanation could be that Notch is delivered to separate endosomal locations via different trafficking pathways when ESCRT-III is depleted. To address this question, we used live imaging of Notch localized on endosomal membranes and investigated the effect of RNAi knockdown of ESCRT-I and ESCRT-III components. When we treated cells with RNAi targeting TSG101, we found that EGFP-tagged Notch remained strictly localized to Cav1-mRFP positively marked endosome membrane regions (Fig. 7 A, Fig. S5 A, and Video 3). As we observed in fixed cells, the endosomes were enlarged and did not contain Cav1-mRFP positive ILVs. Notch-EGFP and Cav1-mRFP comigrated around the endosome surface over a period of several minutes. However, when we treated cells with RNAi targeting the ESCRT-III component Shrub, we observed endosomes in which Notch-EGFP was initially colocalized around the endosome membrane with Cav1-mRFP but over the time course Notch-EGFP and Cav1-mRFP segregated into distinct membrane regions (Fig. 7 B, Fig. S5 B, and Video 4). Similar behavior was observed when Notch-EGFP was coexpressed with VPS4^EQ^ (Fig. 7 C and Video 5). These results therefore indicate an unexpected role of ESCRT complexes in regulating the partitioning of Notch between distinct endosomal membrane domains.

**Video 3.** **Time-lapse live cell spinning disc confocal imaging of transfected N-EGFP (green) and Cav1-mRFP (red) in Tsg101-Knockdown S2R+ cells, showing synchronized localization and dynamics of both puncta on the endosomal limiting membrane.** Representative frames of this movie are shown in Fig. 7 A. Scale bar: 2 μm. Images captured (EGFP: 100 ms, mRFP: 100 ms, SNAP-Cell 647-SiR: 50 ms) every 10 s over 2 μm at 0.34 µm Z-intervals (seven planes) for 10 min at 25°C, average projection of three planes.

**Video 4.** **Time-lapse live cell spinning disc confocal imaging of transfected N-EGFP (green) and Cav1-mRFP (red) in Shrub-Knockdown S2R+ cells, showing lateral migration of Notch compared to Cav1-positive subdomains on endosomal limiting membrane.** Representative frames of this movie are shown in Fig. 7 B. Scale bar: 2 μm. Images captured (EGFP: 100 ms, mRFP: 100 ms, SNAP-Cell 647-SiR: 50 ms) every 10 s over 2 μm at 0.34 µm Z-intervals (7 planes) for 10 min at 25°C, average projection of three planes.

**Video 5.** **Time-lapse live cell spinning disc confocal imaging of transfected N-EGFP (green) and Cav1-mRFP (red) in Vps4^EQ^expressing S2R+ cells, showing lateral migration of Notch compared to Cav1-positive subdomains on endosomal limiting membrane.** Representative frames of this movie are shown in Fig. 7 C. Scale bar: 2 μm. Images captured (EGFP: 100 ms, mRFP: 100 ms, SNAP-Cell 647-SiR: 50 ms) every 10 s over 2 μm at 0.34 µm Z-intervals (seven planes) for 10 min at 25°C, average projection of three planes.

**Figure 8.**
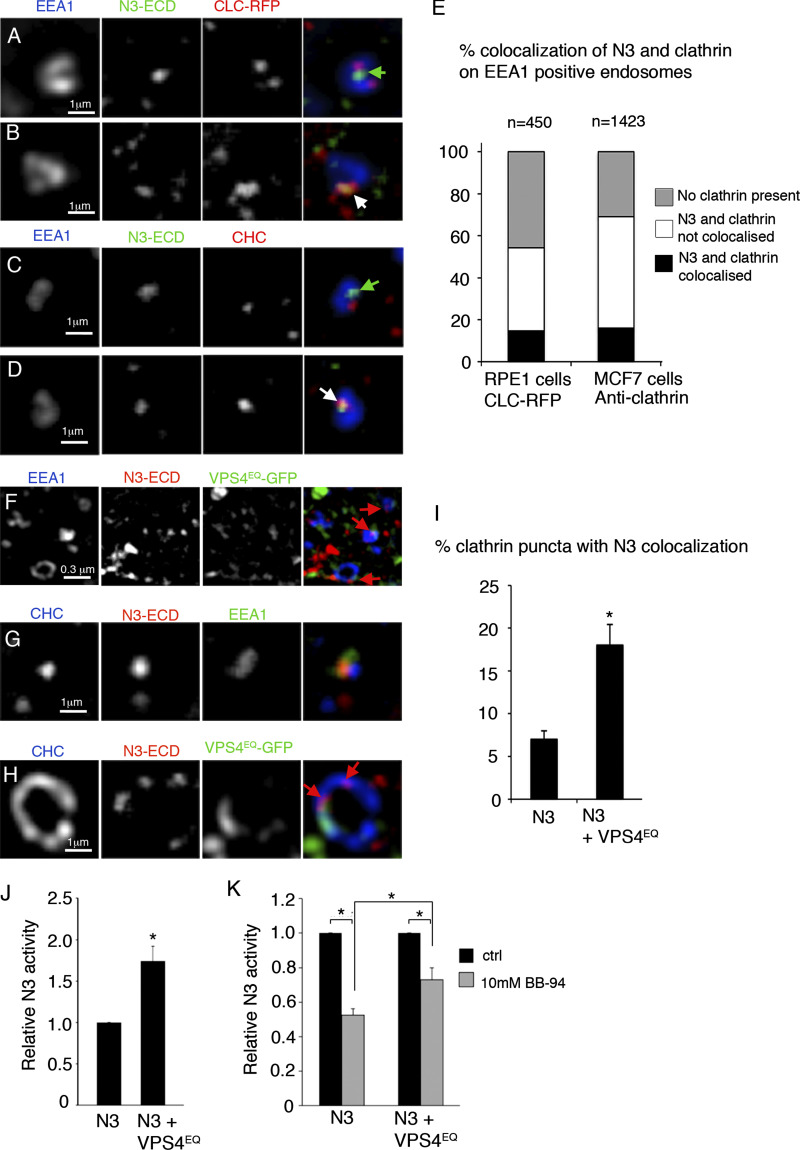
**Human NOTCH3 localizes to different endosomal subdomains and can be activated by VPS4**^**EQ**^**. (A and B)** Localization of human Notch3 expressed in hTERT-RPE-1 cells compared with expressed mRFP-clathrin light chain in EEA1 positive endosome showing either separate localization of Notch and clathrin, green arrow (A) or overlapping localization, white arrow (B). **(C and D)** Localization of endogenous human NOTCH3 in MCF7 cells compared with endogenous clathrin heavy chain (CHC) in EEA1 positive endosome showing either separate localization of NOTCH3 and clathrin, green arrow (C), or overlapping localization, white arrow (D). **(E)** Scoring of % NOTCH3 localization with clathrin in EEA1 endosomes when expressed in htert-RPE-1 cells or endogenous NOTCH3 and clathrin in MCF7 cells. Scoring of overlapping localization was performed on 11 and 16 cells respectively in five optical *Z* sections per cell, total numbers of endosomes scored, are indicated. **(F)** In hTert-RPE1 cells, EGFP-tagged VPS4^EQ^ expression causes enlarged EEA1 positive endosomes with ring-like structures surrounding an enlarged central lumen. Expressed NOTCH3 localized on the perimeter of the organelle (red arrows). VPS4 is localized to sub-regions of the organelle either adjacent to or separate from NOTCH3 and is also localized to EEA1 negative structures. **(G)** NOTCH3 is localized in EEA1-positive endosome, adjacent to the clathrin-positive domain in cells without VPS4^EQ^ expression. **(H)** An enlarged clathrin-positive domain in cell coexpressing NOTCH3 and VPS4^EQ^. NOTCH3 puncta (red arrows) are localized within clathrin marked region, either adjacent or separate from the VPS4^EQ^ occupied region. **(I)** VPS4^EQ^ expression increases % of clathrin stained puncta that also have NOTCH3 localization (*n* = 10 cells scored in Z sections through the cytoplasmic region, a total of 1,505 and 703 clathrin puncta scored, for the presence of NOTCH3, in NOTCH3 and NOTCH3 + VPS4^EQ^ expressing cells, respectively). **(J and K)** VPS4^EQ^ induced the NOTCH3 signal (J) and decreased relative sensitivity to BB-94 (K). * indicates P < 0.05 by two tailed *t* test, error bars are SEM, sample sizes are indicated on figure.

To determine whether the localization of Notch to different endosome membrane microdomains was a shared property with human cells, we investigated the localization of human NOTCH3 since this Notch homolog was previously reported to undergo ligand-independent activation (Xu et al., 2015; Choy et al., 2017). We expressed NOTCH3 in hTert-RPE1 cells and marked endosomes with anti-EEA1, a membrane-tethering factor involved in endosome fusion and maturation (Fig. 8, A, B, and E). We found that around 16% of NOTCH3 puncta, which were located on EEA1 positive endosomes, overlapped with clathrin-RFP when expressed in hTert RPE-1 cells. The remaining NOTCH3 positive endosomes had either separate NOTCH3 and clathrin localization or did not contain clathrin. We also found a similar distribution of endogenous NOTCH3 and clathrin localization on endosomes in MCF7 cells (Fig. 8, C–E), showing that overexpression did not significantly affect the distribution of endosomal NOTCH3 compared to endogenous levels. As with *Drosophila* cells, EGFP-VPS4^EQ^ expression in hTert-RPE1 cells caused enlarged endosomal structures. We also observed increased localization of NOTCH3 in clathrin-positive puncta although we were not able to simultaneously image EEA1, clathrin, NOTCH3, and VPS4^EQ^ all together, and so it is not possible to definitively determine what proportion of these colocalizations are endosomal (Fig. 8, F–I). Where VPS4^EQ^ was present in these clathrin-positive structures, NOTCH3 was adjacent, or separate to VPS4^EQ^, rather than overlapping with it (Fig. 8, F and H). As with *Drosophila* cells, VPS4^EQ^ stimulated human NOTCH3 activity when expressed in hTert-RPE1 cells and reduced the sensitivity of the signal to BB-94 treatment (Fig. 8, J and K). Therefore human NOTCH3, as with *Drosophila* Notch, can switch between different membrane environments associated with different activation mechanisms.

### C-terminal region activating mutations of Notch signal by distinct ligand-independent mechanisms

**Figure 9.**
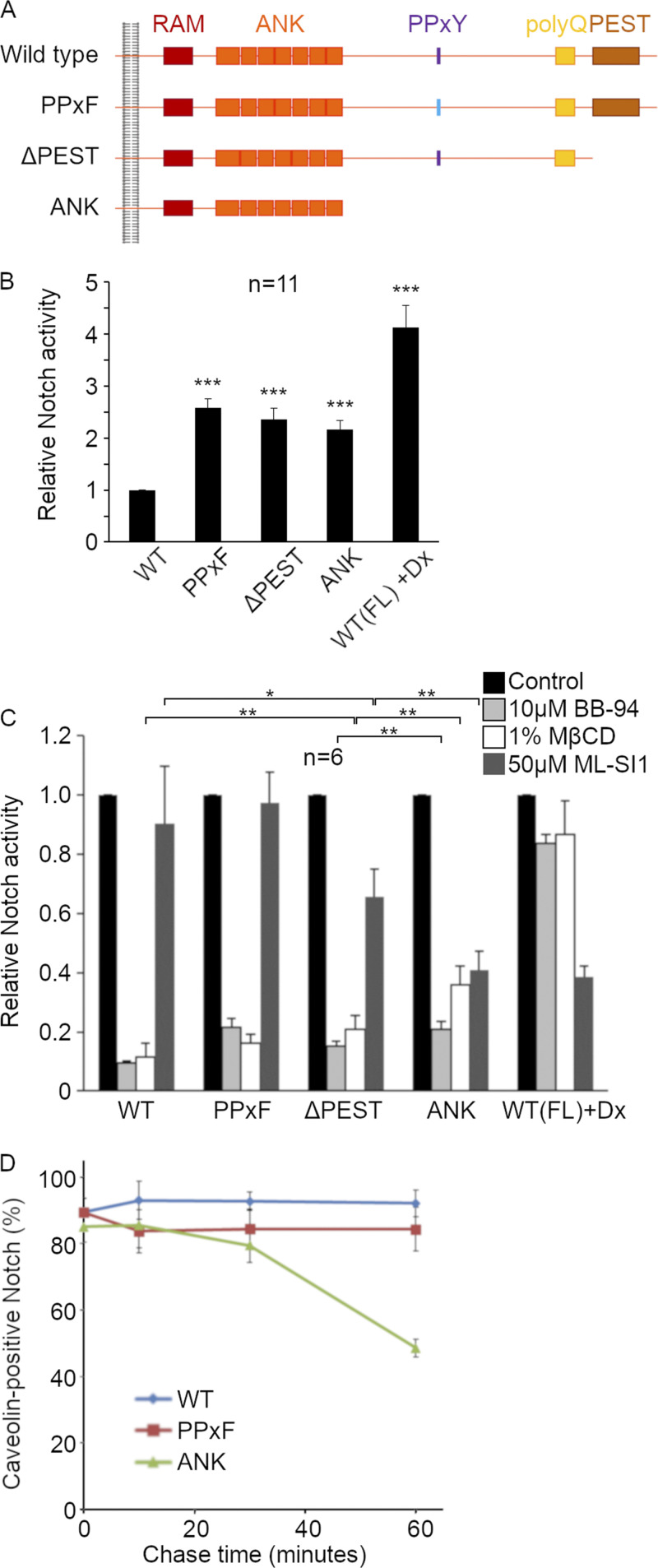
**C-terminal Notch activating mutations signal by alternative mechanisms. (A)** Schematic diagram showing the intracellular domain of *Drosophila* WT Notch and mutant constructs used, PPxF, ΔPEST, and ANK. NB all constructs have intact ECD (not shown). **(B)** Overactivation of the mutant Notch constructs and WT+Dx, analyzed by NRE-luciferase assay in S2 cells. ***P < 0.001 compared to WT Notch. **(C)** Activation of WT Notch and mutant constructs with and without treatment of cells with BB-94, MβCD, or ML-SI1. *, **, and *** indicate P < 0.05, 0.01, and 0.001 by two-tailed *t* test, respectively for comparisons indicated on the graph. Error bars represent SEM. Sample sizes are indicated in figure. **(D)** Time course of colocalization between Notch^ECD^ antibody uptake and Cav-1-mRFP. Notch^ECD^ antibody was labeled for 15 min (pulse), then chased for 0, 10, 30, and 60 min, and the percentage of Notch^ECD^ on Cav1-mRFP positive spots was scored with WT Notch, PPxF, or ANK expressing cells. Data from 3 experimental repeats, with 50–100 puncta scored per repeat, error bars SEM.

Since depletion of ESCRT components perturbs ILV formation and therefore affects endosome morphology, then we wished to determine whether gain-of-function mutations of Notch could also switch between different activating mechanisms, i.e., in conditions where there were no such endosome perturbations. A number of mutations of Notch are known to induce ectopic Notch signaling, including C-terminal region truncations, which have been associated with certain cancers, some genetic syndromes such as Hajdu-Cheney and Lehman syndromes, and gain of function mutant alleles in *Drosophila* (Aster et al., 2017; Weng et al., 2004; Wang et al., 2015; Mašek and Andersson, 2017; Ramain et al., 2001). We therefore generated Notch constructs with differently sized C-terminal truncations, which included the removal of the PEST sequence that affects ICD turnover (Fig. 9 A). We additionally generated a Notch construct, which bears a Tyr to Phe mutation that removes the PPxY motif (Fig. 9 A) that acts as a WW domain binding site, through which Notch associates with Su(dx) (Jennings et al., 2007; Fedoroff et al., 2004). We predicted that the latter mutation would activate Notch through the removal of Su(dx)-dependent downregulation. All constructs showed elevated signaling when expressed in S2 cells compared with wild-type Notch (Fig. 9 B). We probed the mechanistic requirements for the signal activation of each construct using ADAM10 and TRPML inhibitors and cholesterol depletion via MβCD treatment (Fig. 9 C). The Notch^PPxF^ construct had similar activation requirements compared to wild-type Notch and to the stimulated activity resulting from Su(dx) RNAi, being sensitive to ADAM10 inhibitor and cholesterol-depletion and insensitive to TRMPL inhibitor. The activity of Notch^Ank7^, which had an extensive C-terminal deletion from the end of the Ankryin domain region, showed a substantially increased sensitivity to TRPML inhibitor compared to full-length Notch, similar to that exhibited when Notch was activated by coexpression with Deltex (Fig. 9 C). There was also a decrease in sensitivity to cholesterol depletion, while signaling remained sensitive to ADAM10 inhibition. This indicated a hybrid mechanism of activation with features of both the basal and Dx-promoted modes. The activity of Notch^ΔPEST^ was also dependent on ADAM10 and showed intermediate sensitivity to the TRPML inhibitor. The results suggest that removing the C-terminal region of Notch leads to a transfer between membrane environments on the endosome membrane. To investigate this further, we utilized the pulse-chase Notch endocytic uptake assay (Fig. 9 D). We found that Notch^PPxF^ behaved similarly in this assay compared with wild-type Notch, and it remained in Cav1-mRFP-marked endosome locations throughout the time course. However, Notch^Ank^ was found in Cav1-mRFP positive locations early in the time course but the proportion of Notch^Ank^ that colocalized with Cav1-mRFP progressively decreased (Fig. 9 D). Notch^Ank^ therefore behaved similarly when ESCRT-III activity was reduced. The results indicate that different mutant Notch proteins activate ectopically by a variety of mechanisms from different membrane localizations and environments. The dynamic exchange between these environments on the endosome surface determines which mechanism or combination of mechanisms is in operation.

## Discussion

It is becoming increasingly recognized that signaling from intracellular organelle membranes plays an important role in controlling the activity of developmental signaling pathways (Mellman and Yarden, 2013; Schmid, 2017; Gingras et al., 2017). Endosomal membrane compartmentalization into specialized membrane microdomains can provide specific environments, which act as appropriate platforms for signal initiation (Michelet et al., 2009; Hunt et al., 2013; Halls, 2019; Gauthier-Rouvière et al., 2020; Norris and Grant, 2020). However, there is only a limited understanding of how the trafficking of membrane receptors between these different endosomal membrane microdomains is linked to their regulation. We have utilized ligand-independent Notch trafficking and signaling to probe how changes in its endosomal localization between discrete environments on the endosomal surface are regulated and how this affects the choice between different signal activation mechanisms. Previously, clathrin-rich endosomal microdomains have been proposed to act in the transfer of membrane proteins to the lysosome for degradation. Here, we defined two different endosomal microdomains of the late endosome, which were cholesterol-rich or clathrin-rich, and found that each can act as signaling platforms for Notch by different activation mechanisms. We demonstrated the interplay between regulators of endosomal architecture, Notch-interacting trafficking regulators, and function-perturbing mutations to regulate the partitioning of Notch between these two different specific membrane environments. Recruitment of Notch into the clathrin microdomain depended on its ubiquitination by Dx and was associated with a greatly increased efficacy of ligand-independent Notch activation by Dx, whereas recruitment of Notch to the cholesterol-rich microdomain did not depend on the ubiquitin-ligase activity of Su(dx).

In vivo, Notch can be strongly activated by mutation or knockdown of ESCRT-I, II, and III components that normally control cargo selection and transfer to ILVs (Moberg et al., 2005; Vaccari and Bilder, 2005; Vaccari et al., 2009; Schneider et al., 2013; Baeumers et al., 2020). This ectopic activity is assumed to result from the retention of Notch on the endosomal membrane due to depleted ability to transfer to ILVs, but the mechanisms of activation were previously not determined. We recapitulated this activation of Notch in S2 cells using RNAi targeting different ESCRT components. This revealed surprising heterogeneity with regard to which Notch activation mechanism was utilized. This is surprising because the ESCRT-I to III complexes are thought to work together to recruit cargo into ILVs (Raiborg and Stenmark, 2009). We found that depletion of ESCRT-I components resulted in the activation of Notch by the basal ADAM10-dependent mechanism, while disruption of ESCRT-III activity resulted in a significant shift toward a mechanism that is dependent on components involved in late endosome/lysosome fusion. These differences were demonstrated by co-RNAi of endocytic pathway components and by the use of inhibitors of ADAM10 and TRPML. We further found that depletion of ESCRT-I or ESCRT-III activity was also associated with different effects on Notch localization. After TSG101 depletion, Notch remained in Cav1-mRFP marked membrane locations. In contrast, expression of Dx, RNAi knockdown of Shrub, or inhibition by VPS4^EQ^ expression resulted in a shift of Notch away from Cav1-mRFP marked regions to clathrin-positive membrane domains. Thus, removing ESCRT-III function, but not ESCRT-I, was capable of partially bypassing the requirement for Dx for recruitment of Notch to the clathrin-rich endosome membrane domain, with subsequent activation significantly more dependent on lysosomal fusion. We found, using Notch antibody–uptake endocytic assays, that this transfer was a progressive one. ESCRT-III depletion does not affect initial Notch entry into the clathrin-independent pathway or initial localization to Cav1-mRFP marked membrane domains. However, the uptake experiment indicated that continued retention of Notch in this membrane domain depended on ESCRT-III. Depletion of ESCRT-II components caused only a small shift toward the clathrin-dependent route, suggesting that progression through to the later ESCRT-III-dependent steps in the ESCRT pathway was required. Using live imaging of fluorescent protein–tagged components, we were able to directly image segregation of Notch and Cav1-mRFP on the endosome surface in cells treated with Shrub RNAi or expressing VPS4^EQ^, but not in cells treated with RNAi targeting TSG101.

Our results suggested that some ESCRT components play a role in partitioning proteins between different membrane environments. Membrane composition interacts reciprocally with protein/lipid interactions and mechanical forces in driving membrane curvature (Liu et al., 2009). Work on model lipid membranes has shown how artificially induced bud formation is associated with lateral membrane microdomain organization, with cholesterol-enriched regions forming collars around the bud neck that suppress lateral diffusion (Ryu et al., 2014). ESCRT components therefore play a role in defining local membrane composition, which in turn may control local segregation of membrane proteins. For example, using in vitro reconstitution experiments, ESCRT-II complex self-assembly on the membrane was found to require cholesterol and promoted lateral phase separation in artificial membranes (Boura et al., 2012b). ESCRT proteins therefore appear to act as gatekeepers to control lateral membrane protein microdomain localization. Passage of cargo between ESCRT-I/II complexes and ESCRT-III may therefore be associated with a transfer between membrane microdomains, which is revealed when loss of ESCRT-III function prevents ILV formation. Alternatively, ESCRT-III may act to physically prevent Notch movement out of the cholesterol-rich microdomain. ESCRT-III components, including VPS2/Chmp2B and Shrub/SNF7, have previously been proposed to form a movement boundary for cargo, limiting membrane protein diffusion (De Franceschi et al., 2018; Buono et al., 2017). ESCRT-III-dependent segregation of cargo into ILVs is associated with the deubiquitination of cargo, and such diffusion barriers may be required to retain proper cargo localization in the absence of ubiquitin-dependent association with ESCRT-I and II (Frankel and Audhya, 2018). Additionally, some reports have suggested that ESCRT-III complexes assemble at sites of repetitive ILV formation cycles, located at the edge of more stably associated ESCRT-0 positive microdomains. Their presence at this localization may therefore act to control movement between different endosome membrane regions (Frankel, et al., 2017; Buono et al., 2017). Therefore, without these diffusion barrier functions in operation, Notch may be capable of lateral diffusion out of the cholesterol-enriched region to be recruited and retained by the clathrin/ESCRT-0 positive membrane domain, an environment from which it can only subsequently signal through endosomal/lysosomal fusion. These novel roles for ESCRT functions may have wider implications for disease mechanisms as mutations in a number of ESCRT components have been associated with neurodegenerative disorders, microcephaly, and the formation of cataracts (Zivony-Elboum et al., 2012; Lee et al., 2012; Mochida et al., 2012; Shiels et al., 2007; Skibinski et al., 2005), and recent work has identified multisystem defects associated with de novo mutations in VPS4 (Rodger et al., 2020). Emerging evidence also suggests a link between tumor progression and altered expression levels of certain ESCRT components, although there have been few studies of the specific involvement of altered ESCRT function and cancer (Mattissek and Teis, 2014; Manteghi et al., 2016).

The potential for misregulation of transfer between membrane microdomains in disease was further supported by the consequences of activating mutations that remove the C-terminus of the Notch ICD. Similar mutations of Notch have been associated with gain-of-Notch activity across species and are often associated with various cancers in humans (Ramain et al., 2001; Weng et al., 2004; Wang et al., 2015; Mašek and Andersson, 2017). All the truncation mutations that we tested resulted in an increase in activation when expressed in S2 cells compared with wild-type Notch, as did a mutation of the PPXY motif that forms the interaction site with WW domains of Su(dx). Interestingly, however, we found differences in activation requirements of the different Notch mutants. Mutation of the PPXY site resulted in an increase in activity exclusively by the basal, ADAM10-dependent, mechanism and was not affected by TRPML inhibition. Notch^ΔPEST^, which removes the C-terminal region containing the PEST sequence, showed a small but significant increase in sensitivity to TRPML inhibition, while a more extensive C-terminal deletion, that truncates immediately after the Ankyrin domain region, showed a clear shift to TRPML dependency, while remaining sensitive to ADAM10 inhibition, suggesting a hybrid mechanism. Using antibody uptake Notch endocytosis assays, we also observed a transfer of Notch truncation mutants out of Cav1-mRFP marked endosome locations, although less extensive than when cells were treated with RNAi against the ESCRT-III component Shrub. The *Drosophila N*^*mcd*^ alleles (Ramain et al., 2001) are gain-of-function mutants consisting of C-terminal Notch truncations. Interestingly, the gain of activity of these mutants was associated with a Dx-dependent activation mechanism that was suppressed by Dishevelled (Dsh). The latter binds through the C-terminal region and acts as an adaptor protein that recruits and activates Su(dx) to ubiquitinate Notch (Mund et al., 2015). The strength of the gain of function and stage of mutant lethality depended on the size of the truncation with truncations back to the ankyrin domain region showing the strongest effect. Our results cast light on these mutant properties, as in S2 cells we see activation across a range of truncation lengths but a switch in activation mechanism only when truncation extended back to the ankyrin domain region, and this switch may therefore correlate with the increased severity of the mcd alleles with the larger truncations. It is possible that shorter truncations, by removing the Dsh binding site, prevent Su(dx) ubiquitin ligase activation, which depresses basal Notch activity, while larger truncations redirect Notch to activate through late-endosome/lysosome dependent pathway. It will be interesting therefore to explore this network of regulatory interactions further. C-terminal truncation mutants in human cancer have also been found combined with oncogenic Notch mutants in the extracellular negative regulatory domain, which normally protects the S2 cleavage site in the absence of a ligand (Weng et al., 2004). It will be interesting to determine whether this combination of mutants affects the mechanism of activation of different NRR mutants.

**Figure 10.**
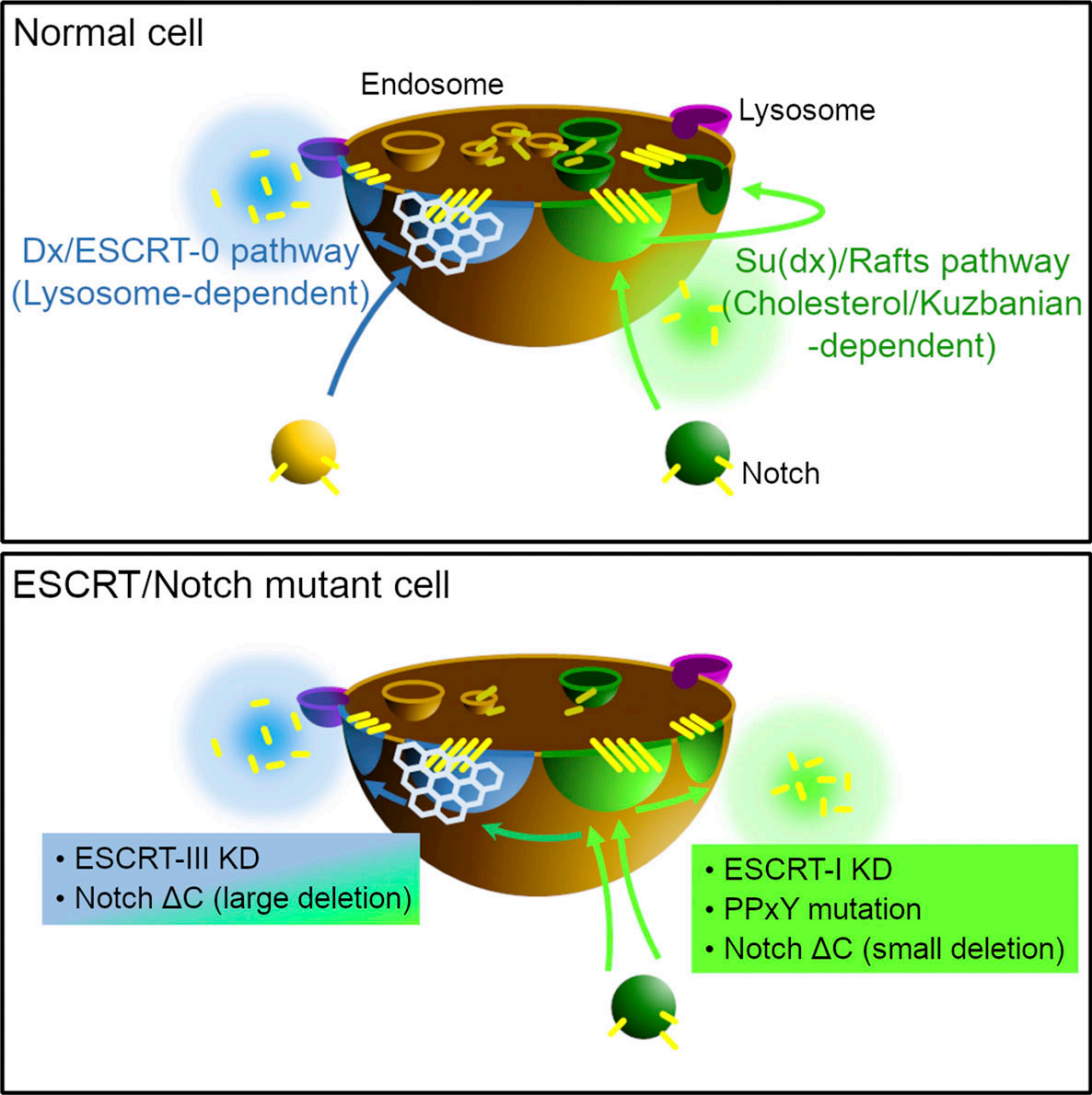
**A schematic model of endosomal microdomains and Notch activation caused by ESCRTs knockdown and Notch mutants.** Dx directs Notch to clathrin (and ESCRT-0)-positive endocytic route and facilitates lysosome-dependent Notch activation while Su(dx) promotes Notch endocytosis via a lipid raft-type membrane subdomain which can activate the signal in ADAM10-dependent manner. ESCRT-I knockdown and mutation in the Notch PPxY motif induce Notch activation through the raft pathway, whereas ESCRT-III knockdown and C-terminal truncations of Notch can switch the route from raft-type pathway to the ADAM10-independent Dx/clathrin pathway.

Overall, our results suggest that partial occupancy of different membrane microdomains may provide multiple mechanisms of Notch activation, with the balance of activity dependent on the combined activity of core ESCRT machinery together with interactions of Notch regulators with the Notch cytoplasmic domain. Defining a mechanism of Notch activation then is more conceptually flexible since different mechanisms can be engaged as Notch is progressively trafficked through different membrane compartments (summarized in Fig. 10). Since human Notch is known to be oncogenic and overactivated in a number of different cancer types and genetic disorders, our findings may be relevant to these disease mechanisms. Indeed, our investigation of human NOTCH3 localization showed that it also to localized to discrete patches on endosome organelles, which could be both within or outside of clathrin-marked regions on the same endosome and associated with different activation mechanisms. It will be interesting to compare these results with other Notch homologs to determine whether there is functional diversity. The knowledge that ectopic activation of Notch can occur by distinct mechanisms or a mixture of such mechanisms will be important when considering how to specifically target such aberrant activity, for example, in Notch-driven tumors, whether arising from mutations of Notch itself or altered regulatory components.

## Materials and methods

### *Drosophila* stocks

All fly stocks were raised on standard *Drosophila* food at 25°C. The following lines were used in this report: *y*^*1*^ *w*^*1*^*, Su(dx)*^*SP*^ (Busseau et al., 1994), *dx*^*152*^ (Fuwa et al., 2006), *car*^*1*^ (Sevrioukov et al., 1999), UAS-shrub RNAi (BDSC 38305), en-Gal4 UAS-myr-mRFP NRE-EGFP (BDSC 30729), and UAS-Tsg101 RNAi (VDRC 23944).

### Cell culture

*Drosophila* S2 cells (Thermo Fisher Scientific) and S2R+ cells (*Drosophila* Genomics Resource Center) were cultured in Schneider’s *Drosophila* medium (Lonza) supplemented with 10% heat-inactivated fetal bovine serum (Thermo Fisher Scientific) and 0.5% Penicillin-Streptomycin (Merck) at 25°C. Effectene (Qiagen) was used for transfection. To generate a pMT-Notch stable cell line, pMT-Notch was cotransfected with pCoHygro construct in S2-R+ cells using Effectene. After the selection of stably transfected cells with 0.3 mg/ml hygromycin B, single clones were isolated in 96-well plates, and then an appropriate line was selected based on immunofluorescence and NRE-luciferase assay.

Human retinal pigmented epithelium (RPE) cells (hTERT-RPE-1) were maintained in Dulbecco’s Modified Eagles Medium F-12 (Merck) supplemented with 1% penicillin-streptomycin (Merck), 1% *L*-glutamine (Thermo Fisher Scientific), and 5% fetal bovine serum (Merck). Cells were fed with fresh medium every 2–3 days and incubated at 37°C in a humidified atmosphere containing 5% CO_2_. Experiments were conducted using passage 10–18 cells. RPE-1 cells were transfected with Genejuice Transfection Reagent (Merck) following the manufacturer’s instruction. The cells were seeded on poly-L-lysine-coated coverslips in six-well plates (Corning) for 24 h at 37°C to reach 60–70% confluency and then transfected with DNA constructs for 2 h. Opti-MEM (Thermo Fisher Scientific) was used as the serum-free medium. The cells were analyzed 24 h after transfection. The hTERT-RPE-1 cell line (Flores-Rodriguez et al., 2011) was a generous gift from Philip Woodman (The University of Manchester, Manchester, UK). Michigan Cancer Foundation-7 (MCF-7) cell line (HTB-22; ATCC) was maintained in Dulbecco’s Modified Eagles Medium (DMEM; Sigma-Aldrich) with low glucose and *L*-glutamine supplemented with 1% penicillin-streptomycin (Sigma-Aldrich) and 5% felt bovine serum (Sigma-Aldrich) in T75 culture flasks (Corning). Cells were fed every 2/3 days with fresh medium and incubated at 37°C in a humidified atmosphere containing 5% CO_2_.

### DNA constructs

pMT-Notch, pMT-Notch^PPxF^, NRE-Firefly Luciferase, pMT-Dx-V5, pMT-HA-Su(dx), pMT-HA-Su(dx)-V5, pMT-Gal4, pUAST-EGFP-GPI, and pMT-Venus-Dx have been previously reported (Shimizu et al., 2014, 2017). pMT-Notch was C-terminally truncated by StuI to make pMT-Notch^ΔPEST^. For pMT-Notch^ANK^ (1–2,148 aa), the Notch intracellular fragment (5,885–6,444 nt) was PCR-amplified with a stop codon TAA and inserted into the corresponding region of pMT-Notch construct. For pMT-Notch-NotI-EGFP, EGFP was inserted at V^2258^, based on position 4 (Couturier et al., 2012). First, a NotI site was introduced at V^2258^ of Notch in pMT-Notch and then EGFP with linkers on both sides was inserted to the NotI site. The resulting product is ---GVSGVPG (Notch 1–2,257) + GGRGGG (linker) + MVSKGEE---GMDELYK (EGFP ORF) + GGGGGR (linker) + PPTNSAA--- (Notch 2259-stop codon). To make pMT-DxΔRF-V5, the ring finger domain (548–600) was removed from pMT-Dx-V5 by PCR-based method. The resulting product is ---LWPNAQP (Dx 1-547) + RS (linker) + GIVYGEK--- (Dx 601-stop codon). Cav1-EGFP (#14433), Cav1-mRFP (#14434), and pEGFP-VPS4-E228Q (#80351) were purchased from Addgene, and required regions were subcloned into pMT/V5-His B vector (RRID:Addgene_17589) to construct expression vectors pMT-Cav1-EGFP, pMT-Cav1-mRFP, and pMT-hVPS4^E228Q^. To construct pMT-Renilla Luciferase, pMT-EGFP-GPI, pMT-SNAP-myc-2xFyve (Keppler et al., 2003), pMT-mTagBFP2-Rab7, pMT-mRFP-clathrin heavy chain, and the corresponding cDNAs were PCR-amplified and then transferred into respective vectors, such as pMT/V5-His B, pMT-EYFP-Rab7, and pMT-mRFP-Rab7. EGFP-clathrin light chain and myr-mRFP were PCR-amplified from genomic DNA of a fly line (Bloomington #7109 and #30729) and subcloned into pMT/V5-His B. C-terminally Flag- and HA-tagged constructs (clathrin heavy chain, Hrs, STAM, Flotillin-2, Atg18a) in pMK33-Flag-HA were purchased from The *Drosophila* Genomics Resource Center. pUAST-EGFP-myc-2xFyve (Wucherpfennig et al., 2003) and pUAST-mCherry-myc-2xFyve (Velichkova et al., 2010) were generous gifts from Hugo J. Bellen (Baylor College of Medicine, Houston, TX, USA) group and Amy A. Kiger group the (University of California, San Diego, CA, USA), respectively. pcDNA3-Notch3 (Wang et al., 2007) was a kind gift from Tao Wang (The University of Manchester, Manchester, UK), and Clc-mRFP (Tagawa et al., 2005) and pMT-EGFP-Actin5c (RRID:Addgene_15312) were purchased from Addgene.

### RNAi

PCR templates for RNAi were from HD2.0 RNAi library (Heidelberg 2) (Horn et al., 2010) and purchased from Sheffield RNAi Screening Facility (The University of Sheffield, Sheffield, UK), with which each dsRNA synthesized using MEGAscript T7 Transcription Kit (Thermo Fisher Scientific) by following the manufacturer’s protocol. The list of dsRNAs used in this manuscript is as follows: hrs (BKN27923), STAM (BKN21390), mvb12 (BKN20544), tsg101 (BKN28961), vps28 (BKN21525), vps37a (BKN29860), vps37b (BKN29819), vps22 (BKN21004), vps25 (BKN23040), vps36 (BKN22165), shrub (BKN28557), vps2 (BKN22197), vps20 (BKN22979), vps24 (BKN27601), vps60 (BKN29120), alix (BKN22801), vps4 (BKN21773), mastermind (BKN30687), shi (BKN21495), rab5 (BKN22991), rab7 (BKN28849), kuzbanian (BKN23747), egghead (BKN27178), glcT-1 (BKN27304), trpml RNAi-1 (BKN20870), trpml RNAi-2 (BKN45122), and dor (BKN22242) (for sequence details https://www.flyrnai.org/up-torr/).

### Real-time quantitative RT-PCR

S2 cells were seeded in six-well plates and treated with dsRNA (20 µg each) for 3 days. RNeasy (Qiagen) and High-Capacity RNA-to-cDNA Kit (Applied Biosystems) were used to purify the total RNA and synthesize cDNA respectively following manufacturers’ instructions. Realtime PCR reactions were performed on StepOnePlus Real-Time PCR System (Applied Biosystems) using Power SYBR Green Master Mix (Applied Biosystems) following manufacturers’ standard protocol. The following primers were used to detect the mRNAs of ESCRT components. 5′-GCA​CTA​GCG​CAT​TGC​AGA​AA-3′ and 5′-GTG​ACT​GGT​GGC​ATT​TTC​TAA​GT-3′ (hrs), 5′-TTC​ACG​GGA​TTT​CGA​GAC​GG-3′ and 5′-GCA​CTT​GGC​GCA​TTT​TCA​GT-3′ (stam), 5′-TTA​TTA​GGC​GCC​GGG​CTG-3′ and 5′-TTC​ATC​GCT​CGG​ATT​CAC​AA-3′ (mvb12), 5′-CCT​GCA​GAG​GTT​CGT​GTT​CA-3′ and 5′-CAT​GGG​TGC​ATT​CTG​GGG​AT-3′ (tsg101), 5′-GTA​CGC​GGA​CTT​CAA​CAA​GC-3′ and 5′-GTC​TGC​GCT​AAA​AGT​AAG​GTG​A-3′ (vps28), 5′-CAA​CCG​GGT​TTG​CCA​ACT​TC-3′ and 5′-TGT​GTC​TGC​GTT​AGA​TCG​GT-3′ (vps37a), 5′-GTC​CTT​GGT​CCT​CAG​CGA​AT-3′ and 5′-TAG​TGC​AGG​CAG​AGA​TGT​GT-3′ (vps37b), 5′-TCA​AGG​ATT​TGG​GCT​GGA​CC-3′ and 5′-GGG​AAA​CCA​ATA​GGC​TGG​CT-3′ (vps22), 5′-CGT​AGT​TAA​TCA​GCA​TGG​CGG-3′ and 5′-CGT​GGG​GCT​GTA​GTG​TAA​AGA-3′ (vps25), 5′-ACG​CGG​GAC​AAC​TTT​ACC​AG-3′ and 5′-TGC​CAC​CTT​GCT​CCT​CGA​TA-3′ (vps36), 5′-AGA​GCC​CAT​CAG​AAT​ATG​GAC​G-3′ and 5′-GGG​TTC​GAA​ATG​GCA​TCG​GA-3′ (shrub), 5′-GCA​CAG​GCC​ATG​AAA​GGT​GTC-3′ and 5′-GGA​TTT​GCG​GGA​GAT​TCA​GC-3′ (vps2), 5′-GAC​AAG​GCG​GTT​CTG​CAA​T-3′ and 5′-ACC​TTG​CTG​TAG​GCA​CTT​CC-3′ (vps20), 5′-AAC​AGT​TAG​TGC​CAT​GGG​CTT​AT-3′ and 5′-CCA​CTC​CTG​CAC​CTG​CTC​TT-3′ (vps24), 5′-ATC​GTC​CCT​GGA​AAG​AGC​AC-3′ and 5′-ATA​CAA​AGC​GTG​CCC​AAG​GT-3′ (vps60), 5′-GTG​CGC​TGG​AGA​CCA​AGA​TA-3′ and 5′-CAA​GGA​GGT​GTG​AGT​CAG​GC-3′ (alix), 5′-GCA​AAA​CCG​AGG​GCT​ACT​CT-3′ and 5′-CAC​TTT​CCT​CAC​GGG​TTC​CA-3′ (vps4), and 5′-TGG​TTT​CCG​GCA​AGC​TTC​AA-3′ and 5′-GCC​ATT​TGT​GCG​ACA​GCT​TA-3′ (rp49).

### Luciferase assay

S2 or S2R+ cells were transfected in a 24-well plate for 24 h and transferred to Nunc 96-well assay plate (Thermo Fisher Scientific) as described previously (Shimizu et al., 2014). Target genes were downregulated by adding 1 µg dsRNA for 48 h followed by additional 24 h with 1 mM CuSO_4_ and inhibitors, including 10 µM BB-94 (APExBIO Technology), 1% MβCD (Merck), and 50 µM ML-SI1 (GW-405833; Cayman Chemical). Dual-Glo Luciferase assay system (Promega) and luminometer (Berthold) were used to quantify Luciferase activity, and Firefly luciferase activity was normalized with Renilla value and analyzed. A small background of luciferase signal present in non-transfected S2-R+ cells was subtracted from data prior to normalization.

RPE-1 cells were transfected in six-well plates with DNA constructs including CBF-Firefly Luciferase (NOTCH3 reporter), 100 ng CMV-Renilla Luciferase (internal control), 0.5 µg pcDNA3-NOTCH3, and 0.5 µg pEGFP-VPS4^EQ^ using Genejuice Transfection Reagent (Merck). Luciferase reporter assay was performed 24 h after transfection using Dual Luciferase Reporter Assay System kit (Promega) following the manufacturer’s protocol. The cells were lysed in 250 µl Passive Lysis buffer and then centrifuged. 20 µl of the lysates were mixed with 50 µl of Luciferase Assay Reagent II and Stop & Glo Reagent in a 96-well assay plate sequentially to measure Firefly and Renilla luciferase activity, respectively. pEGFP-VPS4^EQ^ (Bishop and Woodman, 2000) was a kind gift from Philip Woodman (The University of Manchester, Manchester, UK).

### Ubiquitination assay

S2 cells were transfected with pMT-N-EGFP, pMT-Flag-Ubi, and pMT-Dx-V5 (WT or ΔRF). After 48 h, 1 mM CuSO_4_ was added to induce protein expression, followed by incubation at 18°C for 24 h. The cells were lysed by lysis buffer (50 mM Tris-HCl, pH7.5, 125 mM NaCl, 5% glycerol, 0.5% NP-40, 1.5 mM MgCl2, 1 mM DTT, 1 mM EGTA, 1 mM N-ethyl-maleimide, 10 μM MG132, and Halt Protease and Phosphatase Inhibitor Cocktail [Thermo Fisher Scientific]), and pulled-down with GFP-Trap (ChromoTek). The immunoprecipitated samples and 10% lysates were separated on NuPage 3–8% Tris-Acetate gels (Thermo Fisher Scientific) and transferred to PVDF membranes (Merck). Primary antibodies used for Western blotting were rabbit anti-GFP (50430-2-AP, 1:5,000; Proteintech), mouse anti-Flag (M2, 1:5,000; Merck), mouse anti-V5 (OASA04489, 1:5,000; Aviva System Biology), and mouse anti-Peanut ((Cat# 4C9H4 anti-peanut, RRID:AB_528429, 1:10,000; DSHB), and staining was detected by LI-COR Odyssey imaging system with anti-mouse Alexa Fluor Plus 800 (Cat# A32730, RRID:AB_2633279; Thermo Fisher Scientific) and anti-rabbit Alexa Fluor Plus 680 (Cat# A32734, RRID:AB_2633283; Thermo Fisher Scientific) secondary antibodies, used at 1:10,000.

### Immunofluorescence

#### Immunofluorescence staining of hTert-RPE1 and MCF7 cells

The htert-RPE-1 cells were fixed in 4% formaldehyde (Thermo Fisher Scientific) in PBS for 20 min at room temperature, treated with 0.25% NH_4_Cl, and permeabilized by 0.1% Triton X-100 in PBS for 10 min. The cells were then blocked with 5% NDS (Jackson ImmunoResearch) in PBS for 30 min, followed by primary antibody staining for 1 h and secondary antibody staining for 45 min at room temperature. Primary antibodies used in this study were sheep anti-NOTCH3 ECD (1:500; R&D systems), rabbit anti-NOTCH3 ICD (D11B8, 1:500; Cell Signaling Technology), mouse anti-EEA1 (E9Q6G, 1:200; Cell Signaling Technology), and rabbit anti-clathrin heavy chain (Cat# 4796, RRID:AB_10828486; Cell Signaling Technology, used 1:50).

Images were captured using Volocity (Volocity 3D Image Analysis Software, RRID:SCR_002668) with an Orca-ER digital camera (Hamamatsu) mounted on a M2 fluorescent microscope (Zeiss). Deconvolution was performed with three nearest neighbors using Openlab (Improvision) and processed in Photoshop (RRID:SCR_014199; Adobe Photoshop).

#### Pulse-chase analysis of Notch endocytosis in S2 cells

S2 cells expressing pMT-Notch were grown on poly Lysine-coated coverslips, incubated with C458.2H Notch^ECD^ antibody (1:100; Developmental Studies Hybridoma Bank) for 15 min at 4°C to label surface Notch, washed and incubated in Schneider’s culture medium for 15 min at 25°C to internalize the antibody, and the remaining antibody on the cell surface was masked by unconjugated anti-mouse IgG (1:100; Thermo Fisher Scientific). Notch and the antibody were further endocytosed (for 0, 10, 30, and 60 min) and then fixed by 4% formaldehyde (Thermo Fisher Scientific) in PBS. To process cells on coverslips for immunofluorescence as described before (Shimizu et al., 2014), the cells were washed in PBS three times, permeabilized by 0.2% Triton X-100 in PBS for 15 min, and then blocked in PBS containing 3% skimmed milk for 1 h at room temperature.

The primary antibodies used in this report are rabbit anti-HA (C29F4, 1:500; Cell Signaling Technology), rabbit anti-V5 (LSBio [LifeSpan] Cat# LS-C136565-500, RRID:AB_10915571, 1:500), and mouse anti-Hrs (27-4, 1:10; DSHB). For scoring Notch localization, in each transfection, 50–100 Notch puncta were scored, and transfections were repeated three times.

#### *Drosophila* wing disc staining

Third-instar larvae were dissected in PBS and the wing discs were fixed in PBS containing 4% formaldehyde for 20 min at room temperature. They were subsequently blocked by 100 mM glycine in PBS containing 0.1% Triton X-100 and then incubated with primary antibodies in 4% normal donkey serum in PBS containing 0.1% Triton X-100 at 4°C overnight. The following primary antibodies were used in this study: goat anti-GFP (ab6673, 1:500; Abcam), rat anti-RFP (5F8, 1:500; Chromotek), mouse anti-Hrs (27-4, 1/10; DSHB), and mouse anti Notch extracellular domain (C458.2H, 1:500; DSHB). For NRE-GFP quantification in wing discs, images were opened by Fiji/ImageJ (Schindelin et al., 2012) to measure fluorescence intensities in RFP-negative DV boundary as a standard (NRE_S_), in RFP-positive ventral pouch region as ectopic Notch activation (NRE_E_), and RFP-negative hinge region as a background (NRE_B_). The ectopic Notch signal in each wing disc was presented as (NRE_E_ − NRE_B_)/(NRE_S_ − NRE_B_). Similarly, to quantify the overproliferation induced by ESCRTs knockdown in wing discs, the RFP-positive area of pouch + hinge regions was measured in each wing disc and normalized by the equivalent RFP-negative area.

### Live imaging

S2R+ cells were cultured and transfected in a 24-well plate and transferred to a glass-bottomed culture dish (CELLview; Greiner) with 1 mM CuSO_4_ a day before imaging. The cells were pretreated with ProLong Live Antifade Reagent (Thermo Fisher Scientific) in Schneider’s medium for 30 min, stained with SNAP-Cell 647-SiR (New England Biolabs) for 30 min, and used for live imaging in Schneider’s medium with ProLong Live Antifade up to 3 h. Images were acquired using a CSU-X1 spinning disc confocal (Yokagowa) on a Zeiss Axio-Observer Z1 microscope with a 100×/1.3 Neofluar objective, Evolve EMCCD camera (Photometrics), and motorized XYZ stage (ASI). The 488, 561, and 633 nm lasers were controlled using an AOTF through the laserstack (Intelligent Imaging Innovations [3I]). Slidebook software (3I) was used to capture images (EGFP: 100 ms, mRFP: 100 ms, SNAP-Cell 647-SiR: 50 ms) every 10 s over 2 μm at 0.34 µm Z-intervals (7 planes) for 10 min at 25°C. The images were saved as TIF image sequence files on Slidebook and were converted to hyperstacks to process (average projection of three planes) and analyzed by Fiji/ImageJ. The supplementary movies were generated by converting the TIF hyperstacks to AVI files using Fiji/ImageJ.

### Statistical analysis

All the data were plotted as means with standard errors and the significance was determined using a two-tailed Student’s *t* test. The significance was described as *, **, or *** for P < 0.05, P < 0.01, and P < 0.001, respectively. Data distribution was assumed to be normal, but this was not formally tested. For statistical image analysis of colocalization around endosome perimeter membranes, images were saved as TIFF or LIFF and opened in ImageJ/Fiji to measure and analyze the fluorescence intensity and area. Colocalization of 2D and 1D data was analyzed by Pearson’s correlation coefficient (r) using Coloc2 plugin of ImageJ/Fiji and Microsoft Excel (Microsoft Excel, RRID:SCR_016137), respectively.

### Online supplemental material

Fig. S1 shows the characterization of microdomain markers in S2 cells and their impact on basal Notch signaling. Fig. S2 shows a comparison of expression levels and processing between transfected cells and endogenous Notch from dissected fly tissue. Fig. S3 shows the consequences of ESCRT knockdown on endosomal perimeter size and Cav-mRFP distribution. Fig. S4 shows discrimination between Notch activation mechanisms in transient transfected and stable cell lines. Fig. S5 shows the transition of Notch localization from raft-type membrane microdomain to non-raft domain, comparing TSG101 and shrub knockdown. Video 1 shows time-lapse live cell imaging of N-EGFP and Cav1-mRFP in control S2-R+ cells. Video 2 shows time-lapse live cell imaging of N-EGFP and Cav1-mRFP in Dx-expressing cells. Video 3 shows time-lapse live cell imaging of N-EGFP and Cav1-mRFP in Tsg101-knockdown cells. Video 4 shows time-lapse live cell imaging of N-EGFP and Cav1-mRFP in Shrub-knockdown cells. Video 5 shows time-lapse live cell imaging of N-EGFP and Cav1-mRFP in Vps4EQ-expressing cells. Playback speed: 1 second of video time represents 150 seconds of real time.

## Supplementary Material

SourceData F3 is the source file for Fig. 3.

SourceData FS2 is the source file for Fig. S2.

## Data Availability

The data are available from the corresponding author upon reasonable request.
