# Supplementary figures and images for: Alternative mechanisms of Notch activation by partitioning into distinct endosomal domains
Source: J Cell Biol. 2024 Feb 15;223(5):e202211041. doi: 10.1083/jcb.202211041 (PMC10868400; doi:10.1083/jcb.202211041)

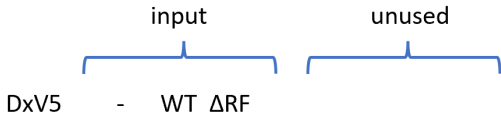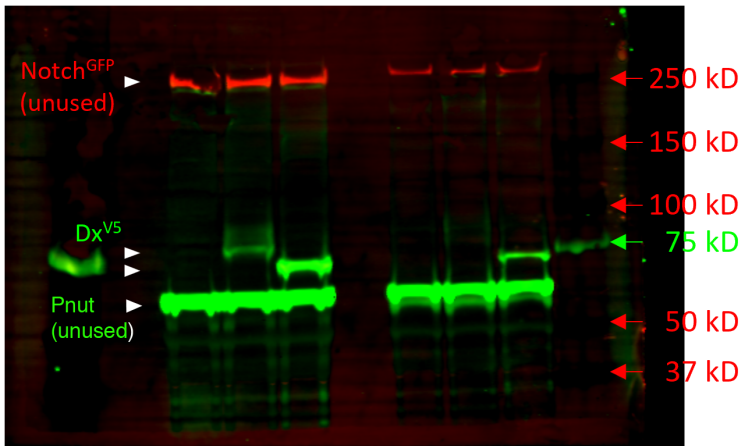

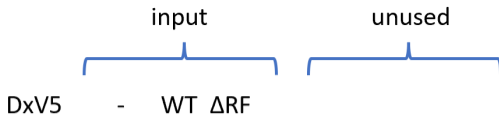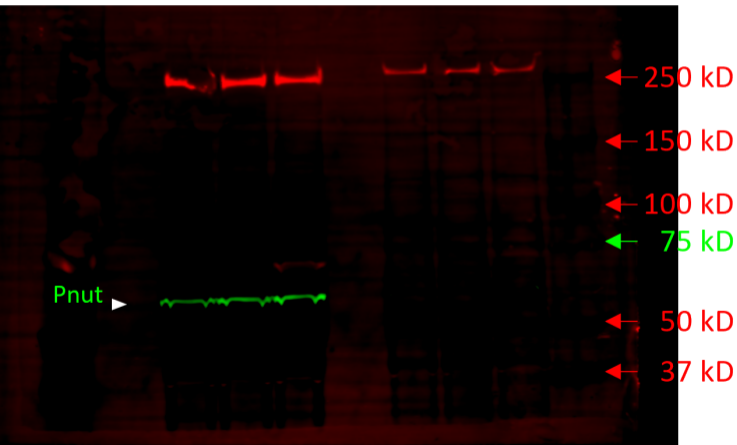

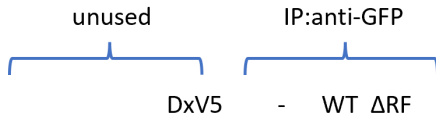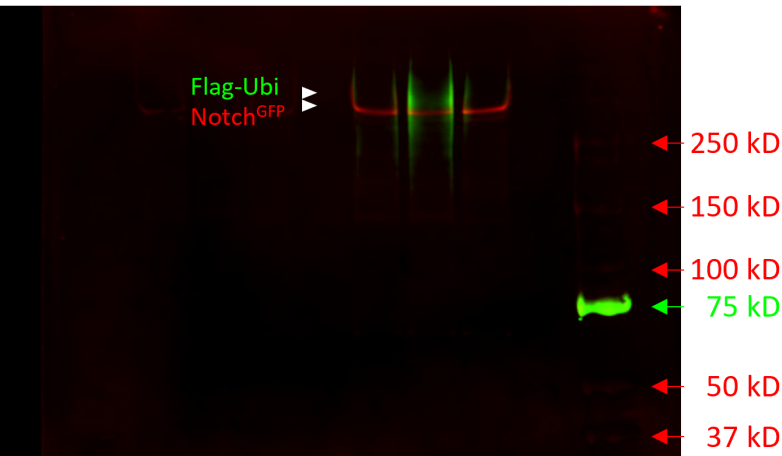

Supplement: SourceData F3 — is the source file for Fig. 3. [file JCB_202211041_SourceDataF3.pdf]

pMT-Notch (ng)    CNS    0    10    20    50    100    200

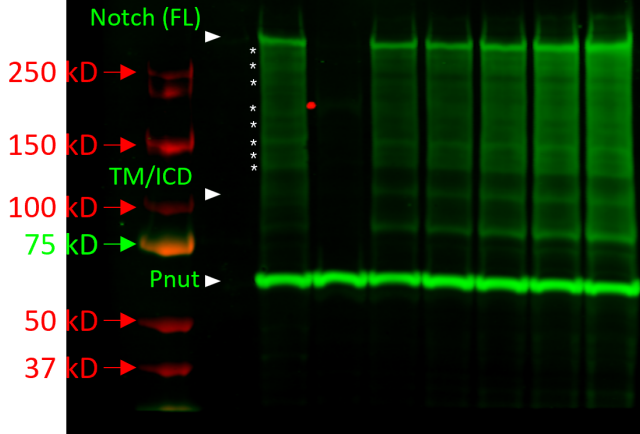

Supplement: SourceData FS2 — is the source file for Fig. S2. [file JCB_202211041_SourceDataFS2.pdf]
